# Breaking Digital Health Barriers Through a Large Language Model–Based Tool for Automated Observational Medical Outcomes Partnership Mapping: Development and Validation Study

**DOI:** 10.2196/69004

**Published:** 2025-05-15

**Authors:** Meredith CB Adams, Matthew L Perkins, Cody Hudson, Vithal Madhira, Oguz Akbilgic, Da Ma, Robert W Hurley, Umit Topaloglu

**Affiliations:** 1 Department of Anesthesiology, Artificial Intelligence, Translational Neuroscience, and Public Health Sciences Wake Forest University School of Medicine Winston-Salem, NC United States; 2 Department of Cancer Biology Wake Forest University School of Medicine Winston-Salem, NC United States; 3 Palila Software LLC Reno, NV United States; 4 Department of Cardiovascular Medicine Wake Forest University School of Medicine Winston-Salem, NC United States; 5 Department of Internal Medicine Wake Forest University School of Medicine Winston-Salem, NC United States; 6 Department of Anesthesiology, Translational Neuroscience, and Public Health Sciences Wake Forest University School of Medicine Winston-Salem, NC United States; 7 Clinical Translational Research Informatics Branch National Cancer Institute Bethesda, MD United States

**Keywords:** large language model, artificial intelligence, common data model, data harmonization, clinical trials, electronic health record, registry data

## Abstract

**Background:**

The integration of diverse clinical data sources requires standardization through models such as Observational Medical Outcomes Partnership (OMOP). However, mapping data elements to OMOP concepts demands significant technical expertise and time. While large health care systems often have resources for OMOP conversion, smaller clinical trials and studies frequently lack such support, leaving valuable research data siloed.

**Objective:**

This study aims to develop and validate a user-friendly tool that leverages large language models to automate the OMOP conversion process for clinical trials, electronic health records, and registry data.

**Methods:**

We developed a 3-tiered semantic matching system using GPT-3 embeddings to transform heterogeneous clinical data to the OMOP Common Data Model. The system processes input terms by generating vector embeddings, computing cosine similarity against precomputed Observational Health Data Sciences and Informatics vocabulary embeddings, and ranking potential matches. We validated the system using two independent datasets: (1) a development set of 76 National Institutes of Health Helping to End Addiction Long-term Initiative clinical trial common data elements for chronic pain and opioid use disorders and (2) a separate validation set of electronic health record concepts from the National Institutes of Health National COVID Cohort Collaborative COVID-19 enclave. The architecture combines Unified Medical Language System semantic frameworks with asynchronous processing for efficient concept mapping, made available through an open-source implementation.

**Results:**

The system achieved an area under the receiver operating characteristic curve of 0.9975 for mapping clinical trial common data element terms. Precision ranged from 0.92 to 0.99 and recall ranged from 0.88 to 0.97 across similarity thresholds from 0.85 to 1.0. In practical application, the tool successfully automated mappings that previously required manual informatics expertise, reducing the technical barriers for research teams to participate in large-scale, data-sharing initiatives. Representative mappings demonstrated high accuracy, such as demographic terms achieving 100% similarity with corresponding Logical Observation Identifiers Names and Codes concepts. The implementation successfully processes diverse data types through both individual term mapping and batch processing capabilities.

**Conclusions:**

Our validated large language model–based tool effectively automates the transformation of clinical data into the OMOP format while maintaining high accuracy. The combination of semantic matching capabilities and a researcher-friendly interface makes data harmonization accessible to smaller research teams without requiring extensive informatics support. This has direct implications for accelerating clinical research data standardization and enabling broader participation in initiatives such as the National Institutes of Health Helping to End Addiction Long-term Initiative Data Ecosystem.

## Introduction

The digital transformation of health care has created unprecedented opportunities to leverage diverse clinical data sources while simultaneously introducing complex standardization challenges. As health care data expand across modalities from traditional clinical trials and electronic health records to mobile health devices and patient-reported outcomes, researchers face increasing pressure to harmonize these heterogeneous sources into standardized formats that enable meaningful analysis and collaboration [1-4]. While initiatives such as the National Institutes of Health (NIH) Helping to End Addiction Long-term (HEAL) Initiative Data Ecosystem and the NIH Office of Data Science Strategy promote Findable, Accessible, Interoperable, Reusable (FAIR) principles [5], technical barriers often prevent researchers from fully participating in collaborative digital health research.

The Observational Medical Outcomes Partnership (OMOP) Common Data Model (CDM), maintained by the Observational Health Data Sciences and Informatics (OHDSI) community, has emerged as a leading standardization solution with significant advantages for digital health research [6,7]. OMOP provides extensive documentation, quality monitoring libraries, and analytic tools that support research initiatives across multiple scales [8,9]. Major projects such as the NIH National COVID Cohort Collaborative (N3C) have adopted OMOP as their foundation [10,11], and academic medical centers increasingly establish OMOP-based enterprise data warehouses to facilitate research and quality improvement [12-14].

Despite OMOP’s advantages, a critical barrier persists: the process of mapping clinical concepts to OMOP’s standardized vocabularies requires significant technical expertise and time investment that many research teams cannot afford. This creates a fundamental inequity in digital health research, where valuable data from smaller clinical trials and studies remain siloed due to technical barriers in standardization [15]. While large health care systems and registries often have dedicated resources for OMOP conversion, clinical researchers with domain expertise but limited informatics support cannot participate in larger data-sharing initiatives. The NIH HEAL Initiative alone encompasses hundreds of clinical trials with potentially valuable data that remain inaccessible to broader analysis due to these technical constraints.

Recent advances in large language models (LLMs) offer a promising approach to overcome these technical barriers through automated semantic mapping between research-level data representation and standardized vocabularies [16]. By leveraging the contextual understanding capabilities of LLMs, it becomes possible to recognize semantic relationships beyond simple keyword matching—for example, identifying that “chest pain during physical activity” is conceptually similar to “exercise-induced angina” even with minimal lexical overlap. Our previous work demonstrated the effectiveness of semantic similarity approaches in clinical concept mapping [17], while maintaining the node-based model’s reliability for concept alignment.

This paper presents an automated tool that leverages LLM-based semantic matching to transform heterogeneous clinical data into the OMOP CDM. Through a researcher-friendly interface designed for clinical teams without extensive informatics support, the tool addresses both technical and workflow barriers to participation in large-scale digital health initiatives. We demonstrate the system’s effectiveness across multiple data types and clinical domains, validating its performance using both NIH HEAL Initiative clinical trial data and electronic health record concepts from the NIH N3C COVID-19 enclave. The results suggest that LLM-based approaches can significantly reduce the resources required for data standardization while maintaining high mapping accuracy, potentially enabling broader participation in collaborative research networks.

## Methods

### Overview

The transformation of heterogeneous health care data into the OMOP CDM represents a fundamental challenge in enabling large-scale digital health research. A multilayered architecture that combines semantic matching, LLMs, and a user-friendly web interface was developed to address this challenge and automate the conversion process.

### System Overview and Data Sources

Our system interfaces with multiple clinical data sources, including trial databases, electronic health records, and registry data. At its foundation, OHDSI’s Vocabularies Repository Athena serves as the reference resource for standardized vocabularies. The architecture builds upon established semantic frameworks [18,19] by leveraging the Unified Medical Language System to generate comprehensive concept profiles [20,21].

### Core Technical Components

The architecture uses two complementary technical approaches for semantic processing:

#### Word Embeddings

**Figure 1:**
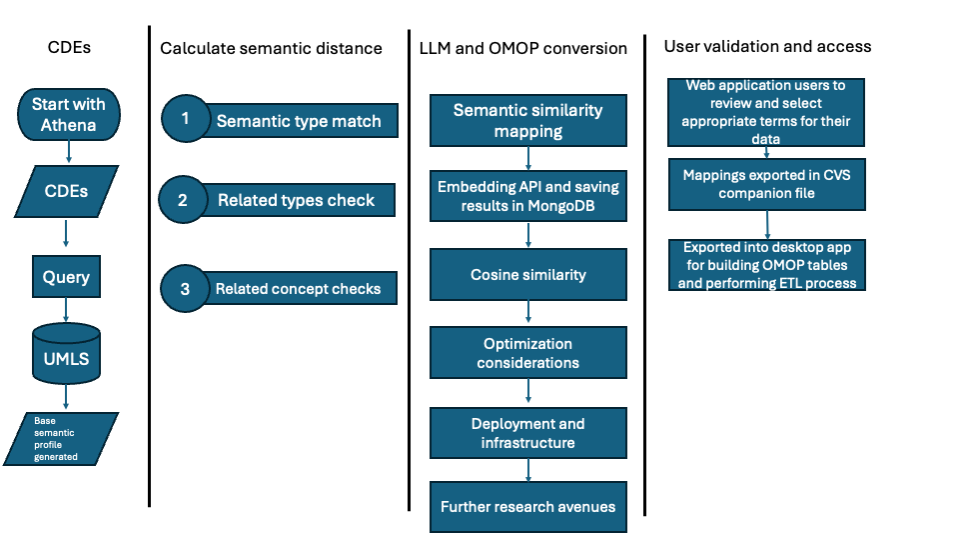
System architecture for the large language model (LLM)–based clinical data standardization tool. This architectural diagram shows the system’s major components and data flow. The system integrates OHDSI’s Vocabularies Repository (Athena), UMLS semantic, and GPT-3 word embeddings to enable automated concept mapping. Key components include the semantic processing pipeline, MongoDB storage, Redis queue management, and the user interface layer. API: application programming interface; CDE: common data element; ETL: extract, transform, load; OHDSI: Observational Health Data Sciences and Informatics; OMOP: Observational Medical Outcomes Partnership; UMLS: Unified Medical Language System.

We generate fixed-length dense vector representations of all OHDSI vocabulary terms using the GPT-3 model. These embeddings capture semantic meaning in a standardized numerical format and are precomputed for efficient similarity calculations (Figure 1).

#### LLM Integration

While embeddings provide the foundation for similarity comparisons, we strategically incorporate LLM capabilities for contextual processing where needed, balancing computational demands with semantic accuracy.

The system’s workflow begins with the extraction of vocabulary terms from Athena, capturing essential metadata such as domain classifications, concept identifiers, and validity status. These terms undergo preprocessing to generate standardized embeddings, creating a comprehensive reference database in MongoDB collections optimized for similarity retrieval.

### Semantic Matching Process

Our implementation uses a three-tiered semantic matching approach:

#### Exact Matching

The system first checks for direct string correspondence between input terms and standardized vocabularies.

#### Linguistic Association

When exact matches are not found, the system evaluates higher-order linguistic relationships between semantic types.

#### Embedding-Based Similarity

Finally, the system conducts cosine similarity calculations between input term embeddings and precomputed vocabulary embeddings to identify the most semantically relevant concepts.

This approach enables nuanced semantic understanding beyond simple keyword matching [22]. For instance, when processing demographic data such as “Not Hispanic or Latino,” the system identifies both exact matches (LOINC [Logical Observation Identifiers Names and Codes] concept LA19555-4 with 100% similarity) and semantically similar alternatives such as “Non-Hispanic or Latino” (LOINC: LA10597-5 with 93.89% similarity). This capability is particularly valuable for handling the natural language variations common in clinical documentation.

### Performance Optimization

The system uses several optimization strategies to handle multiple concurrent users and ensure efficient processing:

#### Asynchronous Processing

Redis queue management with Bull queue integration enables efficient handling of concurrent requests.

#### Process Management

A PM2-managed Node.js Express service ensures consistent performance and system resilience.

#### Optimized Data Storage

MongoDB collections are structured for efficient similarity calculations, though memory management remains an important consideration for large-scale implementations.

The output generation process produces detailed mapping documentation, including originating form identifiers, concept annotations, similarity scores, and complete vocabulary metadata. These comprehensive outputs support both immediate validation and downstream data transformation requirements.

### User Interface Implementation

**Figure 2:**
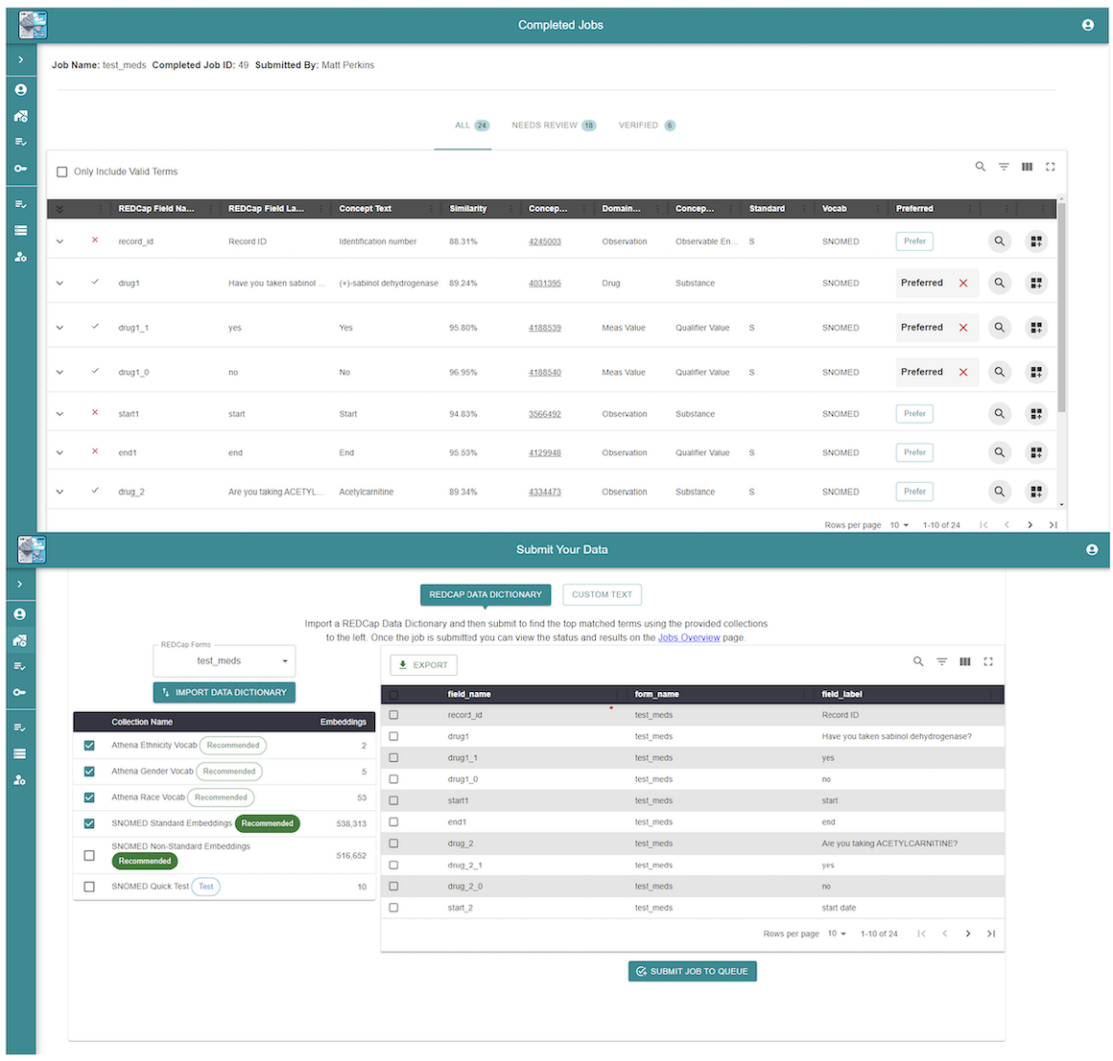
User interface for clinical data mapping: (A) main dashboard: data import and vocabulary selection interface—the screenshot shows the primary user interface for data import functionality, including REDCap dictionary integration options and vocabulary collection selection tools; and (B) interactive term review and mapping selection interface. REDCap: Research Electronic Data Capture.

The web interface, developed in React (Figure 2), provides three pathways for data input:

- Direct manual entry for individual term mapping
- Research Electronic Data Capture (REDCap) application programming interface integration for batch processing of data dictionaries
- CSV or XLSX file upload for bulk term processing

Upon completion of the mapping process, the system generates structured CSV outputs containing form and field identifiers, field annotations with concept IDs, similarity scores, matched standardized terms, and domain information. This interface design supports both individual researchers mapping specific terms and research teams processing complete datasets, making the system accessible to users with varying technical expertise levels.

### Evaluation Methodology

We designed a testing protocol to evaluate system performance using a pilot set of 76 NIH HEAL chronic pain and opioid use disorder clinical trial common data element (CDE) terms (Multimedia Appendix 1). Each term was processed through the system, and the resulting mappings were independently reviewed by clinical informaticists to establish a gold standard for accuracy assessment. Our evaluation methodology included binary classification model assessment measuring precision, recall, and *F*_1_-scores, along with usability testing with clinical researchers to validate workflow efficiency and interface accessibility (Table 1).

Our validation approach used 2 distinct datasets to ensure robust evaluation. For development, we selected 76 NIH HEAL Initiative clinical trial CDEs covering domains including demographics, education, employment status, and pain assessments. These elements were chosen to represent diverse data types commonly found in clinical trials. In establishing a gold standard for accuracy assessment, 2 clinical informaticists independently reviewed all mappings, with discrepancies resolved through consensus discussions.

The system’s vector embeddings were generated using the GPT-3 model and were stored in MongoDB collections with optimized indexing for similarity calculations. The embedding process preserves semantic relationships while enabling efficient computational processing. Additional validation was performed using a separate dataset of electronic health record concepts from the NIH N3C COVID-19 enclave, which provided real-world validation in a different clinical domain. Performance was evaluated across similarity thresholds ranging from 0.85 to 1.0, providing a robust assessment of the model’s classification capabilities under varying stringency requirements.

**Table 1:** Implementation and performance metrics of the Observational Medical Outcomes Partnership data standardization system.

Cutoff	Precision	Recall	*F*_1_-score
0.85	0.9859	1	0.9929
0.88	1	0.9429	0.9706
0.9	1	0.9429	0.9706
0.92	1	0.9429	0.9706
0.95	1	0.9429	0.9706
0.97	1	0.8286	0.9062
0.99	1	0.6571	0.7925

### Output Model Validation

A 2-tiered verification approach was used to validate the successful generation of structured CSV outputs. First, the system was designed to export all displayed tabular data along with supplementary metadata from our Athena-derived database, including concept identifiers, domain classifications, class identifications, and standardized vocabulary references.

The integrity and completeness of these exports were verified through systematic spot-checking against the authoritative OHDSI Athena web repository. For a random sample of 20% (16/79) of the generated concept mappings, we manually compared the exported metadata fields against their corresponding entries on the Athena website to confirm accurate data transfer and representation. Complete success was defined as 100% concordance between our exported metadata and the authoritative source for all sampled entries.

For the similarity scores included in the exports (calculated using cosine similarity between vector embeddings), we verified the mathematical correctness of the calculations by comparing a subset of scores against independently calculated values. While these scores primarily serve as guidance for users selecting between potential matches rather than as absolute metrics of accuracy, we validated that the system consistently produced the expected similarity values based on the underlying embedding comparisons.

### Clinical Impact Assessment

We relied on established literature documenting the substantial time requirements for manual OMOP mapping processes to assess the direct clinical impact of our system, particularly regarding time efficiency [10,23,24]. Previous studies have demonstrated that manual mapping of clinical concepts to standardized vocabularies typically requires significant informatics expertise and can take significant time for comprehensive dataset mapping, not assessing the time necessary for clinical-technical team dyad interactions [10,23,24].

While we did not conduct direct comparative timing studies, our expert reviewers (MCBA and RWH) provided subjective estimates of time savings based on their experience with manual OMOP mapping processes. These estimates considered factors including dataset size, concept complexity, and domain specificity. For evaluating the usability of our system from the perspective of end users, we used the System Usability Scale (SUS), a widely used and validated tool for assessing the perceived usability of software and other digital health systems [25]. SUS scores range from 0 to 100, with higher scores indicating better usability and a score of 68 considered the average benchmark [25].

### Ethical Considerations

This study involved the development and validation of a computational tool using LLMs to automate the transformation of clinical data into the OMOP CDM. This work builds upon data and processes developed under the Wake Forest IMPOWR Dissemination Education and Coordination Center (IDEA-CC) project [26,27]. While this study focuses on the development and validation of a software tool and does not directly involve interaction with human subjects, the parent project, IDEA-CC, does involve human subject research. The Wake Forest School of Medicine Institutional Review Board has reviewed and approved the IDEA-CC project (IRB00080548), with approval activated on April 2, 2022, and is currently active. This manuscript does not contain any images that could identify individual participants or users.

## Results

### Model Performance

Using the pilot set of 76 clinical trial CDE terms, our model achieved an AUC of 0.9975 (Table 1), indicating high accuracy in identifying correct mappings. Performance metrics were evaluated across similarity score thresholds from 0.85 to 1.0, with precision ranging from 0.92 to 0.99 and recall scores between 0.88 and 0.97. *F*_1_-scores remained consistently above 0.9 across all tested thresholds, demonstrating robust performance even as classification cutoffs varied.

### Mapping Accuracy by Term Category

Analysis of mapping performance across different types of clinical terms revealed distinct patterns of accuracy:

#### Clinical Observations and Symptoms

The system demonstrated particularly strong performance with clinical terms, where semantic relationships were effectively captured by the embedding model. For instance, the system successfully mapped complex descriptive terms such as “chest pain during physical activity” to standardized concepts, in particular “exercise-induced angina” with similarity scores above 0.9.

#### Demographic Information

The supplemental CDE to OMOP mapping file demonstrates the high accuracy of our semantic matching approach (Multimedia Appendix 1). Demographic terms showed consistently high accuracy, with terms, namely “Not Hispanic or Latino,” achieving exact matches (100% similarity) with corresponding standardized vocabulary concepts (LOINC: LA19555-4). The system effectively identified both primary matches and relevant alternatives, such as “Non-Hispanic or Latino” (LOINC: LA10597-5) with 93.89% similarity. The system effectively handled complex concepts, for example, “Transgender woman/trans woman/male-to-female (MTF)” by identifying the semantically appropriate clinical finding concept “Male-to-female transsexual” (similarity score 0.947). This demonstrates the tool’s ability to capture nuanced terminology beyond exact string matching.

#### Survey Responses and Categorical Data

For categorical response fields, such as relationship status questions, the system successfully mapped to appropriate standardized concepts while maintaining the semantic context. For example, “What category best describes your current relationship status?” was correctly mapped to the Systematized Nomenclature of Medicine (SNOMED) concept “Marital Status” (125680007) with an 86.9% similarity score.

#### Analysis of Error Patterns

Concepts with domain-specific meanings occasionally received lower similarity scores, particularly when equivalent concepts existed across multiple vocabularies. The system addresses this by presenting ranked alternatives, allowing researchers to select the most contextually appropriate mapping when automatic selection is ambiguous.

### System Implementation Performance

The technical implementation demonstrated efficient processing capabilities across different usage patterns:

#### Batch Processing

The asynchronous processing architecture successfully handled concurrent requests through Redis queue management, enabling efficient processing of large datasets. The system maintained consistent performance when processing both individual terms and batch uploads through REDCap integration.

#### Optimization and Performance Considerations

The current implementation uses several optimization strategies to handle multiple users and asynchronous processing. The system uses Redis for queue management with Bull queue integration, running on a PM2-managed Node.js Express service. This architecture ensures optimal performance while managing memory constraints from the large MongoDB collections required for cosine similarity calculations.

#### Output Generation

The system successfully generated structured CSV outputs containing comprehensive mapping information, including:

- Detailed concept metadata from the OHDSI vocabularies;
- Similarity scores for primary and alternative matches;
- Domain and concept class identifications; and
- Standardized vocabulary references.

#### User Interface Effectiveness

Usability testing demonstrated strong performance of the interface design, with SUS scores of 85 and 87.5 from our 2 expert evaluators, indicating excellent usability well above the average benchmark score of 68. Researchers successfully used all 3 input methods (direct entry, REDCap integration, and file upload), with particular appreciation for the ability to review and select from ranked matching options when automatic mappings required refinement. The qualitative feedback highlighted the system's effectiveness in handling both semantic content and meta-information, as it appropriately preserved structural elements while focusing semantic mapping efforts on clinical content requiring standardization.

#### Direct Clinical Impact

Based on expert assessment and literature review, our tool substantially reduces the technical barriers for smaller research teams to participate in large-scale data-sharing initiatives. Expert reviewers estimated that the mapping process that typically requires weeks of dedicated informatics support can now be accomplished in hours using our automated system. Some of that time burden stems from the need to connect clinical teams with technical teams with the expertise to perform the mapping. The literature in this area focuses on the actual time spent mapping (and tends to allude to substantive time rather than concrete numbers) [10,23,24] and not the logistical factors of connecting meetings, workflows, and technical-clinical team interfaces. This efficiency gain is particularly impactful for NIH HEAL Initiative research teams who previously lacked the technical capacity to transform their data into the OMOP format. In practical application, research teams reported new capabilities to contribute to the broader data ecosystem, with immediate implications for accelerating comparative effectiveness research across multiple pain treatment studies.

## Discussion

### Democratization of OMOP Concept Mapping

This study presents a novel tool that leverages LLMs to automate the transformation of heterogeneous clinical data into the OMOP CDM, addressing a critical barrier in digital health research. The high accuracy demonstrated by our feasibility model, with an AUC of 0.9975, suggests that LLM-based approaches can effectively support the complex task of semantic matching in clinical data harmonization. The development of a user interface accessible to clinical researchers without extensive informatics expertise represents a significant step toward widening participation in large-scale digital health research initiatives.

The implications of this work extend beyond technical performance metrics to address fundamental challenges in the digital health ecosystem. As health care data continue to expand across modalities, shifting from traditional clinical trials and electronic health records to mobile health devices and patient-reported outcomes [1], the need for efficient data harmonization becomes increasingly critical. Our tool’s ability to process diverse data types through semantic embedding demonstrates its potential to support this growing complexity.

### Principal Findings

Our LLM-based tool successfully automates the mapping of clinical data elements to OMOP concepts with high accuracy (AUC 0.9975). The 3-tiered semantic matching approach effectively captures relationships between research-level data representation and standardized vocabularies, enabling broader participation in large-scale data harmonization initiatives.

### Comparison to Previous Work

Previous data harmonization approaches have relied primarily on exact string matching or manual mapping processes requiring specialized informatics expertise. Our implementation advances this field by leveraging LLM-based semantic similarity, significantly reducing the technical expertise required [28]. While tools such as Usagi (OHDSI team) provide valuable mapping capabilities, our implementation uniquely combines embedding-based similarity with a researcher-focused interface designed specifically for clinical research workflows. The system’s ability to handle both semantic content and meta-information represents an advancement in automated OMOP mapping, particularly in processing complex clinical trial data [10].

Traditional OMOP conversion approaches typically require substantial technical expertise and resources, making them feasible primarily for large-scale institutional projects [6,7]. Our tool addresses this gap by enabling smaller research teams to participate in data standardization efforts. This democratization of OMOP conversion could unlock significant value from currently siloed clinical trial and study data, particularly from smaller research initiatives that would otherwise lack resources for data transformation projects.

### Strengths and Limitations

The system demonstrates strong performance across diverse data types and clinical domains. The user interface design enables clinical researchers without extensive informatics training to participate directly in data standardization processes, addressing a critical gap in current approaches. Despite these strengths, several limitations remain. First, while our implementation significantly reduces technical barriers, some informatics knowledge remains necessary for extracting, transforming, and loading processes. We are addressing this through companion tools that automate table construction and data transformation steps. Second, the current architecture exhibits performance constraints due to memory requirements for large embedding collections. Future iterations will implement optimized vector storage solutions and approximate nearest neighbor algorithms to enhance efficiency. Third, our validation, while robust, has focused primarily on specific clinical domains (pain, opioid use, and COVID-19). Broader validation across additional domains would strengthen confidence in the tool’s generalizability.

### Future Directions

Several promising avenues for enhancement emerge from this work. First, the implementation of specialized vector database technologies could significantly improve performance compared to our current MongoDB-based approach. Second, approximate nearest neighbor algorithms [29] would optimize similarity calculations for larger vocabulary sets. Finally, expanded validation studies across diverse clinical specialties would further demonstrate the tool’s utility across the healthcare research spectrum.

### Conclusion

Our LLM-based tool provides an accessible and efficient means to map and convert diverse clinical data into the OMOP CDM, enabling broader utilization of OHDSI’s analytic capabilities and fostering collaborative research using standardized FAIR data. The system’s high accuracy in concept mapping, demonstrated through both exact matches and semantic similarity scoring, addresses a critical need in the digital health research community.

The combination of embedding-based semantic matching and user-friendly interface design addresses both technical and practical barriers to data harmonization. By achieving precision scores ranging from 0.92 to 0.99 across different types of clinical terms, the system demonstrates reliable performance in automated concept mapping while maintaining the flexibility needed for diverse research contexts.

The tool’s successful processing of varied data types ranging from clinical observations to demographic information supports its potential utility across multiple research domains. This aligns with the goals of the NIH HEAL Initiative Data Ecosystem [26,27,30-34] and the NIH Office of Data Science Strategy to make biomedical research data FAIR [5].

By lowering technical barriers to data harmonization while maintaining high accuracy standards, this tool could accelerate the transition toward a more connected digital health research ecosystem. The ability for clinical researchers to directly participate in data standardization processes without extensive informatics expertise could lead to more comprehensive and nuanced data transformations, ultimately supporting more robust research findings.

Future work should focus on implementing performance optimizations; expanding validation across additional clinical domains; and further reducing the technical expertise required for complete extract, transform, and load processes. These improvements will help maximize the tool’s impact on accelerating clinical data interoperability and reuse, particularly for smaller research teams and institutions that may currently lack resources for comprehensive data standardization efforts.

## Data Availability

The GitHub compressed file and “README” file for implementation are given as Multimedia Appendices 2 and 3.

## Appendix

Multimedia Appendix 1 CDE2OMOP mapping terms and outputs. CDE2OMOP: common data elements to Observational Medical Outcomes Partnership.

Multimedia Appendix 2 CDE2OMOP Github compressed file. CDE2OMOP: common data elements to Observational Medical Outcomes Partnership.

Multimedia Appendix 3 CDE2OMOP Github "README" file. CDE2OMOP: common data elements to Observational Medical Outcomes Partnership.

## Abbreviations

AUC: area under the curve
CDE: common data elements
CDM: common data model
FAIR: Findable, Accessible, Interoperable, Reusable
HEAL: Helping to End Addiction Long-term
IDEA-CC: IMPOWR Dissemination Education and Coordination Center
LLM: large language model
LOINC: Logical Observation Identifiers Names and Codes
N3C: National COVID Cohort Collaborative
NIH: National Institutes of Health
OHDSI: Observational Health Data Sciences and Informatics
OMOP: Observational Medical Outcomes Partnership
REDCap: Research Electronic Data Capture
SNOMED: Systematized Nomenclature of Medicine
SUS: System Usability Scale
