# Supplementary material for: Breaking Digital Health Barriers Through a Large Language Model–Based Tool for Automated Observational Medical Outcomes Partnership Mapping: Development and Validation Study
Source: J Med Internet Res. 2025 May 15;27:e69004. doi: 10.2196/69004 (PMC12123247; doi:10.2196/69004)
Supplement: Multimedia Appendix 1 [file jmir_v27i1e69004_app1.docx]

Supplemental File 1. Common Data Elements to OMOP Mapping Terms and Outputs

This supplemental file contains the dataset used for validation of our LLM-based OMOP mapping tool. The file includes 76 NIH HEAL Initiative clinical trial Common Data Elements (CDEs) covering domains such as demographics, education, employment status, and pain assessments. For each term, the file presents the input source term, the mapped OMOP standardized concept, similarity scores, concept identifiers, and domain classifications.

The file demonstrates the system's semantic matching capabilities across various clinical terminology types. Key features illustrated in this dataset include:

1. Input clinical trial terms in their original format
2. OMOP standardized concept matches with corresponding similarity scores
3. Complete metadata from the OHDSI vocabularies including concept IDs, domains, and vocabulary sources
4. Primary and alternative matches when multiple potential concepts were identified
5. Detailed classification information for each mapping

| **Field Label** | **Concept Name** | **Similarity** | **Concept ID** | **Domain ID** | **Concept Class ID** | **Vocab** |
| --- | --- | --- | --- | --- | --- | --- |
| Date of enrollment | Date of admission | 0.9355487980019515 | 4161014 | Observation | Observable Entity | SNOMED |
|  | Trial enrollment date | 0.9079307791881412 | 1015470 | Observation | LOINC Component | LOINC |
|  | Trial enrollment date | 0.9079307791881412 | 42528933 | Observation | Clinical Observation | LOINC |
|  | Admission date | 0.9069281629468825 | 1031920 | Observation | LOINC Component | LOINC |
|  | Admission date | 0.9069281629468825 | 3040041 | Observation | Clinical Observation | LOINC |
|  | Date of entry | 0.8987319200411924 | 1032183 | Observation | LOINC Component | LOINC |
|  | Date of entry | 0.8987319200411924 | 3030697 | Observation | Survey | LOINC |
|  | Date of inpatient admission | 0.8907786915645319 | 1030387 | Observation | LOINC Component | LOINC |
|  | Date of event | 0.8888269123274369 | 4208903 | Observation | Observable Entity | SNOMED |
|  | Date received | 0.8865320880246332 | 1030395 | Observation | LOINC Component | LOINC |
| What is your birth date? | Birth date | 0.9123161258177713 | 3022007 | Observation | Clinical Observation | LOINC |
|  | Birth date | 0.9123161258177713 | 40782825 | Observation | LOINC Component | LOINC |
|  | Date of birth | 0.9036528756198688 | 4083587 | Observation | Observable Entity | SNOMED |
|  | Birth time | 0.8863856807277443 | 1034671 | Observation | LOINC Component | LOINC |
|  | Birth time | 0.8863856807277443 | 40760826 | Observation | Clinical Observation | LOINC |
|  | Birth year | 0.87356367 | 1014772 | Observation | LOINC Component | LOINC |
|  | Birth year | 0.8726062236984123 | 21494070 | Observation | Survey | LOINC |
|  | Birth detail | 0.8722376769161778 | 4014291 | Observation | Observable Entity | SNOMED |
|  | Birth date Family member | 0.8716526970021234 | 3051549 | Observation | Survey | LOINC |
|  | Able to remember own date of birth | 0.8705504222217116 | 4114901 | Condition | Clinical Finding | SNOMED |
| Are you of Hispanic, Latino, or Spanish origin? | Hispanic, latino-a, or spanish origin | 0.9154060696053131 | 1010401 | Observation | LOINC Component | LOINC |
|  | Hispanic, latino-a, or spanish origin | 0.9154060696053131 | 42868351 | Observation | Clinical Observation | LOINC |
|  | Hispanic or Latino | 0.9055950281855273 | 38003563 | Ethnicity | Ethnicity | Ethnicity |
|  | Hispanic or Latino | 0.9054224606461223 | 1034436 | Observation | LOINC Component | LOINC |
|  | Hispanic or Latino | 0.9054224606461223 | 40759172 | Observation | Clinical Observation | LOINC |
|  | Hispanic or Latino | 0.9054224606461223 | 45882065 | Meas Value | Answer | LOINC |
|  | Hispanic origin | 0.9004721292741154 | 1030598 | Observation | LOINC Component | LOINC |
|  | Hispanic origin | 0.9004721292741154 | 40759211 | Observation | Clinical Observation | LOINC |
|  | Yes, another Hispanic, Latino, or Spanish origin | 0.8990971471323741 | 1034971 | Meas Value | Answer | LOINC |
|  | Yes, another Hispanic, Latino-a, or Spanish origin | 0.896512088 | 45885113 | Meas Value | Answer | LOINC |
| Not Hispanic or Latino | Not Hispanic or Latino | 0.9999982686715049 | 38003564 | Ethnicity | Ethnicity | Ethnicity |
|  | Not Hispanic or Latino | 0.9999940341217844 | 45885168 | Meas Value | Answer | LOINC |
|  | Not hispanic or latino | 0.965461773 | 1009417 | Observation | LOINC Component | LOINC |
|  | Non-Hispanic or Latino | 0.9388666051498957 | 45884900 | Meas Value | Answer | LOINC |
|  | Hispanic or Latino | 0.9219124546657784 | 38003563 | Ethnicity | Ethnicity | Ethnicity |
|  | Hispanic or Latino | 0.9218087947229416 | 1034436 | Observation | LOINC Component | LOINC |
|  | Hispanic or Latino | 0.9218087947229416 | 40759172 | Observation | Clinical Observation | LOINC |
|  | Hispanic or Latino | 0.9218087947229416 | 45882065 | Meas Value | Answer | LOINC |
|  | White/Not Hispanic | 0.9121454414483442 | 45880774 | Meas Value | Answer | LOINC |
|  | Not hispanic or latino [#] Census tract | 0.9096033639094996 | 40770357 | Observation | Clinical Observation | LOINC |
| Yes Hispanic or Latino | Hispanic or Latino | 0.9668793978026045 | 38003563 | Ethnicity | Ethnicity | Ethnicity |
|  | Hispanic or Latino | 0.9668442092006934 | 1034436 | Observation | LOINC Component | LOINC |
|  | Hispanic or Latino | 0.9668442092006934 | 40759172 | Observation | Clinical Observation | LOINC |
|  | Hispanic or Latino | 0.9668442092006934 | 45882065 | Meas Value | Answer | LOINC |
|  | Non-Hispanic or Latino | 0.9383121129107098 | 45884900 | Meas Value | Answer | LOINC |
|  | Hispanic or Latino (Other Hispanic) | 0.9329122 | 36309774 | Meas Value | Answer | LOINC |
|  | Hispanic | 0.9318424455523977 | 45876999 | Meas Value | Answer | LOINC |
|  | Hispanic/Latino | 0.9304231481898599 | 1620311 | Meas Value | Answer | LOINC |
|  | Other Hispanic/Latino/ Spanish | 0.927515427 | 45882063 | Meas Value | Answer | LOINC |
|  | Latino | 0.9270154975804796 | 45883615 | Meas Value | Answer | LOINC |
| Unknown | Unknown | 0.9999961875583503 | 4129922 | Observation | Qualifier Value | SNOMED |
|  | Unknown | 0.999995092 | 8552 | Race | Race | Race |
|  | Unknown | 0.9999731078404569 | 45877986 | Meas Value | Answer | LOINC |
|  | UNKNOWN | 0.9513602916498343 | 8551 | Gender | Gender | Gender |
|  | Uknown | 0.9224628824791372 | 45881441 | Meas Value | Answer | LOINC |
|  | Not known | 0.907670103 | 45884595 | Meas Value | Answer | LOINC |
|  | Reason unknown | 0.9073702764294879 | 45882805 | Meas Value | Answer | LOINC |
|  | Unidentified | 0.9046531446976757 | 4321411 | Observation | Qualifier Value | SNOMED |
|  | Other unknown | 0.9027144885325459 | 45879342 | Meas Value | Answer | LOINC |
|  | Unidentified | 0.9026903379476107 | 36308643 | Meas Value | Answer | LOINC |
| Decline to answer | Decline to answer | 0.9999925327260036 | 36210418 | Meas Value | Answer | LOINC |
|  | Refused to answer | 0.9370247965484715 | 36032578 | Meas Value | Answer | LOINC |
|  | I prefer not to answer | 0.901969638 | 1177221 | Meas Value | Answer | LOINC |
|  | Other/Unknown/Refuse To Answer | 0.9004032453991047 | 45877393 | Meas Value | Answer | LOINC |
|  | Unable to answer or no response | 0.8917808566723734 | 45885006 | Meas Value | Answer | LOINC |
|  | Unable to answer | 0.8877913838553885 | 45881125 | Meas Value | Answer | LOINC |
|  | I choose not to answer this question | 0.8847731840934208 | 37079361 | Meas Value | Answer | LOINC |
|  | Declined to provide information about financial circumstances | 0.8767060610125501 | 3657444 | Observation | Context-dependent | SNOMED |
|  | Patient declined to answer family origin questionnaire | 0.8758965029918906 | 44805141 | Observation | Context-dependent | SNOMED |
|  | Declined/Participant doesn't know | 0.871838122 | 21498124 | Meas Value | Answer | LOINC |
| What is your race? | Your race | 0.9330468812918724 | 45883946 | Meas Value | Answer | LOINC |
|  | Race or ethnicity | 0.8966328732780697 | 1033012 | Observation | LOINC Component | LOINC |
|  | Race or ethnicity | 0.8966328732780697 | 3050381 | Observation | Clinical Observation | LOINC |
|  | Multiple races | 0.8763292134418473 | 36310364 | Meas Value | Answer | LOINC |
|  | Other Race | 0.8695928886309093 | 8522 | Race | Race | Race |
|  | Race or ethnicity panel | 0.8669424559968305 | 1013075 | Observation | LOINC Component | LOINC |
|  | More than one race | 0.8663013845185877 | 45880900 | Meas Value | Answer | LOINC |
|  | Race | 0.8661868344010543 | 4013886 | Observation | Observable Entity | SNOMED |
|  | Race | 0.8659004604086088 | 1030008 | Observation | LOINC Component | LOINC |
|  | Race | 0.8659004604086088 | 1032799 | Observation | LOINC System | LOINC |
| White | White | 1.0000000000000033 | 45877987 | Meas Value | Answer | LOINC |
|  | White | 0.9999771852969935 | 8527 | Race | Race | Race |
|  | Yellow | 0.8922657664250124 | 45881930 | Meas Value | Answer | LOINC |
|  | Black | 0.8883644903059289 | 1009438 | Observation | LOINC Component | LOINC |
|  | Black | 0.8883583927604229 | 38003598 | Race | Race | Race |
|  | Black | 0.8882637553187107 | 45876489 | Meas Value | Answer | LOINC |
|  | Purple | 0.8783309864511005 | 35627103 | Observation | Qualifier Value | SNOMED |
|  | Purple | 0.8777924972440253 | 45883951 | Meas Value | Answer | LOINC |
|  | Brown | 0.8772558708976756 | 45884360 | Meas Value | Answer | LOINC |
|  | Pink | 0.8746935673486511 | 45883297 | Meas Value | Answer | LOINC |
| Black/African American | Black/African American | 0.999988804 | 46237778 | Meas Value | Answer | LOINC |
|  | Black or African American | 0.973252006 | 8516 | Race | Race | Race |
|  | Black or African American | 0.973199426 | 45877988 | Meas Value | Answer | LOINC |
|  | African American | 0.9690525210040326 | 36033877 | Observation | LOINC Component | LOINC |
|  | African American | 0.9690525065673491 | 38003599 | Race | Race | Race |
|  | Black or African-American | 0.9684213780968303 | 45883683 | Meas Value | Answer | LOINC |
|  | African-American | 0.9683411399778532 | 45880773 | Meas Value | Answer | LOINC |
|  | Black or african american alone | 0.9179296366800254 | 1009419 | Observation | LOINC Component | LOINC |
|  | African | 0.9178733832653534 | 38003600 | Race | Race | Race |
|  | Hispanic, black | 0.907556856 | 4214410 | Observation | Observable Entity | SNOMED |
| American Indian or Alaska Native | American Indian or Alaska Native | 1.0000000000000022 | 45877442 | Meas Value | Answer | LOINC |
|  | American Indian or Alaska Native | 1.0000000000000022 | 45880297 | Meas Value | Answer | LOINC |
|  | American Indian or Alaska Native | 0.9999940770309659 | 8657 | Race | Race | Race |
|  | American Indian/Alaskan Native | 0.9662711299042888 | 37079234 | Meas Value | Answer | LOINC |
|  | American Indian | 0.9314942898013352 | 45879207 | Meas Value | Answer | LOINC |
|  | American Indian | 0.9314561160334417 | 38003572 | Race | Race | Race |
|  | American indian and alaska native alone | 0.9213273897064997 | 1009420 | Observation | LOINC Component | LOINC |
|  | American Indian, Aleutian, or Eskimo (includes all indigenous populations of the Western hemisphere) | 0.9144594841256386 | 45882400 | Meas Value | Answer | LOINC |
|  | Alaska Native | 0.9128488166373625 | 38003573 | Race | Race | Race |
|  | Alaska Native | 0.9127856578327591 | 45876637 | Meas Value | Answer | LOINC |
| Asian | Asian | 1 | 45879439 | Meas Value | Answer | LOINC |
|  | Asian | 0.9999863737199848 | 8515 | Race | Race | Race |
|  | Asian-American | 0.9036855388981476 | 45882950 | Meas Value | Answer | LOINC |
|  | Japanese | 0.893989888 | 38003584 | Race | Race | Race |
|  | Japanese | 0.893408458 | 45882243 | Meas Value | Answer | LOINC |
|  | Asian people | 0.8924125191709888 | 44803808 | Observation | Qualifier Value | SNOMED |
|  | Asia | 0.8906857679493941 | 4071831 | Observation | Location | SNOMED |
|  | Asian Indian | 0.8849911429630005 | 38003574 | Race | Race | Race |
|  | Other Asian | 0.8838237230109541 | 45881625 | Meas Value | Answer | LOINC |
|  | Thai | 0.8832356708068813 | 38003591 | Race | Race | Race |
| Native Hawaiian or Other Pacific Islander | Native Hawaiian or Other Pacific Islander | 0.9999991366202099 | 45878240 | Meas Value | Answer | LOINC |
|  | Native Hawaiian or Other Pacific Islander | 0.9999889205347926 | 8557 | Race | Race | Race |
|  | Native Hawaiian or Pacific Islander | 0.9884557320398973 | 45877443 | Meas Value | Answer | LOINC |
|  | Samoan or Native Hawaiian | 0.9335561551783149 | 1990223 | Meas Value | Answer | LOINC |
|  | Native Hawaiian | 0.9300268892684783 | 45884422 | Meas Value | Answer | LOINC |
|  | Pacific Islander | 0.9282766787729765 | 37079126 | Meas Value | Answer | LOINC |
|  | Other Pacific Islander | 0.9278204966532444 | 38003613 | Race | Race | Race |
|  | Other Pacific Islander | 0.9277166549553256 | 45883142 | Meas Value | Answer | LOINC |
|  | #NAME? | 0.9269010871418376 | 45882062 | Meas Value | Answer | LOINC |
|  | Native hawaiian and other pacific islander alone | 0.9250316122763353 | 1009422 | Observation | LOINC Component | LOINC |
| Some other race | Other Race | 0.9461192049936089 | 8522 | Race | Race | Race |
|  | Some other race alone | 0.9414030037285079 | 1009423 | Observation | LOINC Component | LOINC |
|  | Some Other Race (specify) | 0.9399204561185337 | 45882402 | Meas Value | Answer | LOINC |
|  | More than one race | 0.9181232159841806 | 45880900 | Meas Value | Answer | LOINC |
|  | Multiple races | 0.9145645928565851 | 36310364 | Meas Value | Answer | LOINC |
|  | Your race | 0.8887769540182382 | 45883946 | Meas Value | Answer | LOINC |
|  | Some other race alone [#] Census tract | 0.8853375153729385 | 40770363 | Observation | Clinical Observation | LOINC |
|  | Race or ethnicity | 0.884098514 | 1033012 | Observation | LOINC Component | LOINC |
|  | Race or ethnicity | 0.884098514 | 3050381 | Observation | Clinical Observation | LOINC |
|  | Race | 0.8725909030393969 | 4013886 | Observation | Observable Entity | SNOMED |
| Unknown | Unknown | 1.0000000000000013 | 45877986 | Meas Value | Answer | LOINC |
|  | Unknown | 0.9999683042382487 | 4129922 | Observation | Qualifier Value | SNOMED |
|  | Unknown | 0.9999653677343708 | 8552 | Race | Race | Race |
|  | UNKNOWN | 0.9512359806288099 | 8551 | Gender | Gender | Gender |
|  | Uknown | 0.9222203919141385 | 45881441 | Meas Value | Answer | LOINC |
|  | Not known | 0.9075393731443372 | 45884595 | Meas Value | Answer | LOINC |
|  | Reason unknown | 0.9071284757133405 | 45882805 | Meas Value | Answer | LOINC |
|  | Unidentified | 0.9046672331885074 | 4321411 | Observation | Qualifier Value | SNOMED |
|  | Unidentified | 0.9027285510176166 | 36308643 | Meas Value | Answer | LOINC |
|  | Unidentified | 0.9027285510176166 | 37041797 | Observation | LOINC Component | LOINC |
| Prefer not to answer | I prefer not to answer | 0.9719876457389129 | 1177221 | Meas Value | Answer | LOINC |
|  | Prefer not to say | 0.9382847263902695 | 36311156 | Meas Value | Answer | LOINC |
|  | Decline to answer | 0.9287031242549465 | 36210418 | Meas Value | Answer | LOINC |
|  | Refused to answer | 0.91642402 | 36032578 | Meas Value | Answer | LOINC |
|  | I choose not to answer this question | 0.9154130555902008 | 37079361 | Meas Value | Answer | LOINC |
|  | No - This question does not apply to me/I prefer not to answer | 0.9058708138354414 | 1621154 | Meas Value | Answer | LOINC |
|  | Unable to answer | 0.8986322951148931 | 45881125 | Meas Value | Answer | LOINC |
|  | Other/Unknown/Refuse To Answer | 0.8970410725628549 | 45877393 | Meas Value | Answer | LOINC |
|  | Unable to answer or no response | 0.8833838251597624 | 45885006 | Meas Value | Answer | LOINC |
|  | Don't know/refused | 0.861506706 | 45876763 | Meas Value | Answer | LOINC |
| Some other race (SPECIFY) | Some Other Race (specify) | 0.9762872046284894 | 45882402 | Meas Value | Answer | LOINC |
|  | Other Race | 0.9212818232730743 | 8522 | Race | Race | Race |
|  | Some other race alone | 0.9087056730593327 | 1009423 | Observation | LOINC Component | LOINC |
|  | Other (specify) | 0.8986646717107281 | 45883155 | Meas Value | Answer | LOINC |
|  | More than one race | 0.8915635478783717 | 45880900 | Meas Value | Answer | LOINC |
|  | Multiple races | 0.8903927753447098 | 36310364 | Meas Value | Answer | LOINC |
|  | Some other race alone [#] Census tract | 0.8753384299364194 | 40770363 | Observation | Clinical Observation | LOINC |
|  | Other, specify | 0.8747452009746155 | 45883153 | Meas Value | Answer | LOINC |
|  | Race or ethnicity | 0.8714068430585599 | 1033012 | Observation | LOINC Component | LOINC |
|  | Race or ethnicity | 0.8714068430585599 | 3050381 | Observation | Clinical Observation | LOINC |
| Gender Identity - Do you think of yourself as . . . | Gender identity | 0.8883480581442069 | 1013447 | Observation | LOINC Component | LOINC |
|  | Gender identity | 0.8883480581442069 | 46235215 | Observation | Clinical Observation | LOINC |
|  | Gender identity finding | 0.8564246467886938 | 4110772 | Condition | Clinical Finding | SNOMED |
|  | Identifies as female gender | 0.8527613814423933 | 765761 | Condition | Clinical Finding | SNOMED |
|  | Identifies as male gender | 0.8509348463329999 | 763768 | Condition | Clinical Finding | SNOMED |
|  | Gender identity disorder | 0.8500434182274887 | 4338512 | Condition | Clinical Finding | SNOMED |
|  | Gender identity disorder of adolescence | 0.8453975949561363 | 432306 | Condition | Clinical Finding | SNOMED |
|  | Identifies as non-conforming gender | 0.8442044426818582 | 763767 | Condition | Clinical Finding | SNOMED |
|  | Identifies as male | 0.8434400990857648 | 36308665 | Meas Value | Answer | LOINC |
|  | Identifies as female | 0.840321863 | 36307702 | Meas Value | Answer | LOINC |
| Male | Male | 1.000000000000001 | 45880669 | Meas Value | Answer | LOINC |
|  | Male | 0.9846515270942493 | 442985 | Condition | Clinical Finding | SNOMED |
|  | Female | 0.9260033498127233 | 45878463 | Meas Value | Answer | LOINC |
|  | Female | 0.9242703119988765 | 442986 | Condition | Clinical Finding | SNOMED |
|  | Adult | 0.9195623538145277 | 4046779 | Observation | Social Context | SNOMED |
|  | Adult | 0.9193536013832927 | 45878201 | Meas Value | Answer | LOINC |
|  | Gender | 0.9114891366033051 | 4135376 | Observation | Observable Entity | SNOMED |
|  | Gender | 0.9111005327854105 | 1028876 | Observation | LOINC Component | LOINC |
|  | Woman | 0.894009561 | 4075501 | Observation | Social Context | SNOMED |
|  | MALE | 0.8916116340791127 | 8507 | Gender | Gender | Gender |
| Female | Female | 1 | 45878463 | Meas Value | Answer | LOINC |
|  | Female | 0.9926225667644762 | 442986 | Condition | Clinical Finding | SNOMED |
|  | Woman | 0.9577041707743005 | 4075501 | Observation | Social Context | SNOMED |
|  | Male | 0.9260033498127233 | 45880669 | Meas Value | Answer | LOINC |
|  | Male | 0.9229947926929272 | 442985 | Condition | Clinical Finding | SNOMED |
|  | Girlfriend | 0.9172661821327961 | 4309206 | Observation | Social Context | SNOMED |
|  | Gender | 0.9107349759594227 | 4135376 | Observation | Observable Entity | SNOMED |
|  | Gender | 0.9103088957270861 | 1028876 | Observation | LOINC Component | LOINC |
|  | Adult | 0.904948596 | 4046779 | Observation | Social Context | SNOMED |
|  | Adult | 0.9046572665853769 | 45878201 | Meas Value | Answer | LOINC |
| Transgender man/trans man/female-to-male (FTM) | Male-to-female transsexual | 0.9367760853365071 | 4248372 | Condition | Clinical Finding | SNOMED |
|  | Male-to-female transsexual | 0.9366089997529005 | 36309787 | Meas Value | Answer | LOINC |
|  | Female-to-male transsexual | 0.9322515556072248 | 4234364 | Condition | Clinical Finding | SNOMED |
|  | Female-to-male transsexual | 0.9319981880103236 | 36309198 | Meas Value | Answer | LOINC |
|  | Transsexual | 0.9141098448039723 | 4231241 | Condition | Clinical Finding | SNOMED |
|  | Transsexual | 0.9140072603402399 | 45882199 | Meas Value | Answer | LOINC |
|  | Male to female transsexual person on hormone therapy | 0.9057291702949348 | 37017838 | Condition | Clinical Finding | SNOMED |
|  | Female to male transsexual person on hormone therapy | 0.9044915651018348 | 37017839 | Condition | Clinical Finding | SNOMED |
|  | Previously heterosexual transsexual | 0.8972435268344702 | 43021158 | Condition | Clinical Finding | SNOMED |
|  | Intersex surgery, male to female | 0.8943514729046156 | 4201284 | Procedure | Procedure | SNOMED |
| Transgender woman/trans woman/male-to-female (MTF) | Male-to-female transsexual | 0.9471604190923102 | 4248372 | Condition | Clinical Finding | SNOMED |
|  | Male-to-female transsexual | 0.9470198352224277 | 36309787 | Meas Value | Answer | LOINC |
|  | Female-to-male transsexual | 0.9441099411227546 | 4234364 | Condition | Clinical Finding | SNOMED |
|  | Female-to-male transsexual | 0.9440166256705065 | 36309198 | Meas Value | Answer | LOINC |
|  | Transsexual | 0.9165284541098861 | 4231241 | Condition | Clinical Finding | SNOMED |
|  | Transsexual | 0.9164052172694723 | 45882199 | Meas Value | Answer | LOINC |
|  | Male to female transsexual person on hormone therapy | 0.90915967 | 37017838 | Condition | Clinical Finding | SNOMED |
|  | Female to male transsexual person on hormone therapy | 0.9085268782172872 | 37017839 | Condition | Clinical Finding | SNOMED |
|  | Previously heterosexual transsexual | 0.896912364 | 43021158 | Condition | Clinical Finding | SNOMED |
|  | Intersex surgery, male to female | 0.89508197 | 4201284 | Procedure | Procedure | SNOMED |
| Genderqueer | Gender identity | 0.9042290825312683 | 1013447 | Observation | LOINC Component | LOINC |
|  | Gender identity | 0.9042290825312683 | 46235215 | Observation | Clinical Observation | LOINC |
|  | Intersex | 0.8926416840547401 | 46273637 | Condition | Clinical Finding | SNOMED |
|  | Gender dysphoria | 0.8881912931884216 | 4246663 | Condition | Clinical Finding | SNOMED |
|  | Identifies as non-conforming gender | 0.8866227538595501 | 763767 | Condition | Clinical Finding | SNOMED |
|  | Gender identity disorder | 0.8801086426609692 | 4338512 | Condition | Clinical Finding | SNOMED |
|  | Intersexuality | 0.8796137575595302 | 4048723 | Condition | Clinical Finding | SNOMED |
|  | Transsexual | 0.8795586826199081 | 4231241 | Condition | Clinical Finding | SNOMED |
|  | Transsexual | 0.8792860901457155 | 45882199 | Meas Value | Answer | LOINC |
|  | Female-to-male transsexual | 0.8756995749241434 | 4234364 | Condition | Clinical Finding | SNOMED |
| Nonbinary | Intersex | 0.8751588631906657 | 46273637 | Condition | Clinical Finding | SNOMED |
|  | Bisexual | 0.873923171 | 4170582 | Observation | Clinical Finding | SNOMED |
|  | Bisexual | 0.8737028737599123 | 36307527 | Meas Value | Answer | LOINC |
|  | Identifies as non-conforming gender | 0.8655627035358378 | 763767 | Condition | Clinical Finding | SNOMED |
|  | Gender identity | 0.8638453433544394 | 1013447 | Observation | LOINC Component | LOINC |
|  | Gender identity | 0.8638453433544394 | 46235215 | Observation | Clinical Observation | LOINC |
|  | Intersexuality | 0.8635933715300705 | 4048723 | Condition | Clinical Finding | SNOMED |
|  | Sexually attracted to neither male nor female sex | 0.8603281238262818 | 35623015 | Observation | Clinical Finding | SNOMED |
|  | Indeterminate sex | 0.8601776417612875 | 46270485 | Condition | Clinical Finding | SNOMED |
|  | Indeterminate sex | 0.8601776417612875 | 4295345 | Spec Anatomic Site | Body Structure | SNOMED |
| Agender | Gender identity | 0.8998931868024146 | 1013447 | Observation | LOINC Component | LOINC |
|  | Gender identity | 0.8998931868024146 | 46235215 | Observation | Clinical Observation | LOINC |
|  | Intersex | 0.8855377974986023 | 46273637 | Condition | Clinical Finding | SNOMED |
|  | Gender identity disorder | 0.8754939834881257 | 4338512 | Condition | Clinical Finding | SNOMED |
|  | Intersexuality | 0.8733748351389038 | 4048723 | Condition | Clinical Finding | SNOMED |
|  | Gender dysphoria | 0.872888959 | 4246663 | Condition | Clinical Finding | SNOMED |
|  | Gender | 0.8705747521123469 | 4135376 | Observation | Observable Entity | SNOMED |
|  | Gender | 0.8700211886427238 | 1028876 | Observation | LOINC Component | LOINC |
|  | Identifies as non-conforming gender | 0.8689949580842738 | 763767 | Condition | Clinical Finding | SNOMED |
|  | Transsexual | 0.864646335 | 4231241 | Condition | Clinical Finding | SNOMED |
| Gender Fluid | Gender identity | 0.9352214681233895 | 1013447 | Observation | LOINC Component | LOINC |
|  | Gender identity | 0.9352214681233895 | 46235215 | Observation | Clinical Observation | LOINC |
|  | Gender dysphoria | 0.9157359048245826 | 4246663 | Condition | Clinical Finding | SNOMED |
|  | Gender identity disorder | 0.8969313285817444 | 4338512 | Condition | Clinical Finding | SNOMED |
|  | Intersex | 0.8966811474119593 | 46273637 | Condition | Clinical Finding | SNOMED |
|  | Transsexual | 0.8962400241905549 | 4231241 | Condition | Clinical Finding | SNOMED |
|  | Transsexual | 0.8959996386572097 | 45882199 | Meas Value | Answer | LOINC |
|  | Gender identity finding | 0.8911460019345088 | 4110772 | Condition | Clinical Finding | SNOMED |
|  | Gender | 0.8895019939545077 | 4135376 | Observation | Observable Entity | SNOMED |
|  | Identifies as non-conforming gender | 0.8892746171062474 | 763767 | Condition | Clinical Finding | SNOMED |
| Some other gender | Your gender | 0.8800727735030796 | 45883294 | Meas Value | Answer | LOINC |
|  | Other (hermaphrodite) | 0.878290905 | 45881548 | Meas Value | Answer | LOINC |
|  | Gender | 0.8699304857406973 | 4135376 | Observation | Observable Entity | SNOMED |
|  | Gender | 0.8692532654891916 | 1028876 | Observation | LOINC Component | LOINC |
|  | Masculinized female | 0.8683715517552221 | 4154780 | Condition | Clinical Finding | SNOMED |
|  | Female structure | 0.8662671997736997 | 4031903 | Spec Anatomic Site | Body Structure | SNOMED |
|  | Identifies as female gender | 0.8648927896046886 | 765761 | Condition | Clinical Finding | SNOMED |
|  | Female homosexual | 0.8603359014217179 | 4230492 | Observation | Clinical Finding | SNOMED |
|  | Gender identity | 0.8600538736007621 | 1013447 | Observation | LOINC Component | LOINC |
|  | Gender identity | 0.8600538736007621 | 46235215 | Observation | Clinical Observation | LOINC |
| Prefer not to answer | I prefer not to answer | 0.9719876457389129 | 1177221 | Meas Value | Answer | LOINC |
|  | Prefer not to say | 0.9382847263902695 | 36311156 | Meas Value | Answer | LOINC |
|  | Decline to answer | 0.9287031242549465 | 36210418 | Meas Value | Answer | LOINC |
|  | Refused to answer | 0.91642402 | 36032578 | Meas Value | Answer | LOINC |
|  | I choose not to answer this question | 0.9154130555902008 | 37079361 | Meas Value | Answer | LOINC |
|  | No - This question does not apply to me/I prefer not to answer | 0.9058708138354414 | 1621154 | Meas Value | Answer | LOINC |
|  | Unable to answer | 0.8986322951148931 | 45881125 | Meas Value | Answer | LOINC |
|  | Other/Unknown/Refuse To Answer | 0.8970410725628549 | 45877393 | Meas Value | Answer | LOINC |
|  | Unable to answer or no response | 0.8833838251597624 | 45885006 | Meas Value | Answer | LOINC |
|  | Don't know/refused | 0.861506706 | 45876763 | Meas Value | Answer | LOINC |
| Please specify for gender category | Gender | 0.8467315026082074 | 4135376 | Observation | Observable Entity | SNOMED |
|  | Gender | 0.8460472938274449 | 1028876 | Observation | LOINC Component | LOINC |
|  | Other: PLEASE SPECIFY | 0.8453040961376125 | 45884046 | Meas Value | Answer | LOINC |
|  | Identifies as female gender | 0.842637792 | 765761 | Condition | Clinical Finding | SNOMED |
|  | Identifies as male gender | 0.8422073287512063 | 763768 | Condition | Clinical Finding | SNOMED |
|  | Your gender | 0.8411752427400799 | 45883294 | Meas Value | Answer | LOINC |
|  | Age category | 0.8398874848675205 | 1619416 | Observation | LOINC Component | LOINC |
|  | Age/sex details on computer | 0.8369509632071614 | 4081596 | Observation | Clinical Finding | SNOMED |
|  | Other: specify | 0.8336535309439946 | 1621139 | Meas Value | Answer | LOINC |
|  | Sex of Volunteer | 0.8318785242430614 | 37021278 | Measurement | Clinical Observation | LOINC |
| What was your sex assigned at birth? | Sex assigned at birth | 0.9081879292232871 | 46235213 | Observation | Clinical Observation | LOINC |
|  | Sex assigned^at birth | 0.8767188952746281 | 1023297 | Observation | LOINC Component | LOINC |
|  | Sex assigned | 0.8584751039512574 | 1013495 | Observation | LOINC Component | LOINC |
|  | Sex of baby at delivery | 0.8582610993060225 | 4092760 | Observation | Observable Entity | SNOMED |
|  | Your gender | 0.8538075488689538 | 45883294 | Meas Value | Answer | LOINC |
|  | Sex of baby | 0.8464046965104969 | 4149371 | Observation | Observable Entity | SNOMED |
|  | Was the baby a boy or a girl | 0.8451208423481018 | 1006903 | Observation | LOINC Component | LOINC |
|  | Gender identity | 0.8437388093888443 | 1013447 | Observation | LOINC Component | LOINC |
|  | Gender identity | 0.8437388093888443 | 46235215 | Observation | Clinical Observation | LOINC |
|  | Fetal sex | 0.8409839620785039 | 37028031 | Observation | LOINC Component | LOINC |
| Male | Male | 0.9999975620047199 | 442985 | Condition | Clinical Finding | SNOMED |
|  | Male | 0.9847108565398419 | 45880669 | Meas Value | Answer | LOINC |
|  | Female | 0.9248217560676947 | 442986 | Condition | Clinical Finding | SNOMED |
|  | Female | 0.9230222690184662 | 45878463 | Meas Value | Answer | LOINC |
|  | Adult | 0.9126738383988664 | 4046779 | Observation | Social Context | SNOMED |
|  | Adult | 0.9124433255998733 | 45878201 | Meas Value | Answer | LOINC |
|  | Gender | 0.9050290181308546 | 4135376 | Observation | Observable Entity | SNOMED |
|  | Gender | 0.9045856111999462 | 1028876 | Observation | LOINC Component | LOINC |
|  | Woman | 0.8915870991911035 | 4075501 | Observation | Social Context | SNOMED |
|  | Baby male | 0.8814588885439769 | 4015423 | Condition | Clinical Finding | SNOMED |
| Female | Female | 0.9983719503740777 | 45878463 | Meas Value | Answer | LOINC |
|  | Female | 0.9948246453640001 | 442986 | Condition | Clinical Finding | SNOMED |
|  | Woman | 0.9558993078284405 | 4075501 | Observation | Social Context | SNOMED |
|  | Male | 0.9249910436480073 | 45880669 | Meas Value | Answer | LOINC |
|  | Male | 0.9244566365448325 | 442985 | Condition | Clinical Finding | SNOMED |
|  | Girlfriend | 0.9153140027841133 | 4309206 | Observation | Social Context | SNOMED |
|  | Gender | 0.9098658838608615 | 4135376 | Observation | Observable Entity | SNOMED |
|  | Gender | 0.9093968492067255 | 1028876 | Observation | LOINC Component | LOINC |
|  | Adult | 0.9023975584536312 | 4046779 | Observation | Social Context | SNOMED |
|  | Adult | 0.9020982975966846 | 45878201 | Meas Value | Answer | LOINC |
| Intersex | Intersex | 0.999995424 | 46273637 | Condition | Clinical Finding | SNOMED |
|  | Intersexuality | 0.9586948143318913 | 4048723 | Condition | Clinical Finding | SNOMED |
|  | Intersex surgery | 0.9273539853304584 | 4089389 | Procedure | Procedure | SNOMED |
|  | Transsexual | 0.915158981 | 4231241 | Condition | Clinical Finding | SNOMED |
|  | Transsexual | 0.9149930739908968 | 45882199 | Meas Value | Answer | LOINC |
|  | Gender identity | 0.9006352621126514 | 1013447 | Observation | LOINC Component | LOINC |
|  | Gender identity | 0.9006352621126514 | 46235215 | Observation | Clinical Observation | LOINC |
|  | Intersex surgery, female to male | 0.8962827937086336 | 4179532 | Procedure | Procedure | SNOMED |
|  | Intersex surgery, male to female | 0.895939305 | 4201284 | Procedure | Procedure | SNOMED |
|  | Gender dysphoria | 0.8954048417651487 | 4246663 | Condition | Clinical Finding | SNOMED |
| Unknown | Unknown | 0.9999964706147041 | 4129922 | Observation | Qualifier Value | SNOMED |
|  | Unknown | 0.9999948749827011 | 8552 | Race | Race | Race |
|  | Unknown | 0.999972924 | 45877986 | Meas Value | Answer | LOINC |
|  | UNKNOWN | 0.9513721115075349 | 8551 | Gender | Gender | Gender |
|  | Uknown | 0.9224719931146478 | 45881441 | Meas Value | Answer | LOINC |
|  | Not known | 0.9077028396585436 | 45884595 | Meas Value | Answer | LOINC |
|  | Reason unknown | 0.907341532 | 45882805 | Meas Value | Answer | LOINC |
|  | Unidentified | 0.9046408724264378 | 4321411 | Observation | Qualifier Value | SNOMED |
|  | Other unknown | 0.9027395828833237 | 45879342 | Meas Value | Answer | LOINC |
|  | Unidentified | 0.9026783049156142 | 36308643 | Meas Value | Answer | LOINC |
| Other | Other | 0.9999981351790052 | 9177 | Observation | Qualifier Value | SNOMED |
|  | Other | 0.9999894770429053 | 1032802 | Observation | LOINC Component | LOINC |
|  | Other | 0.9999894770429053 | 3040314 | Observation | Survey | LOINC |
|  | Other | 0.9999894770429053 | 21498861 | Meas Value | Answer | LOINC |
|  | Other | 0.9999894770429053 | 45878142 | Meas Value | Answer | LOINC |
|  | Others | 0.960667126 | 40776267 | Observation | LOINC Component | LOINC |
|  | Others | 0.9605792114951915 | 1620997 | Meas Value | Answer | LOINC |
|  | OTHER | 0.9324530474755288 | 8521 | Gender | Gender | Gender |
|  | All other | 0.9141522660900455 | 36032719 | Meas Value | Answer | LOINC |
|  | Else | 0.9090487397551286 | 45879273 | Meas Value | Answer | LOINC |
| Prefer not to answer | I prefer not to answer | 0.9720143346730313 | 1177221 | Meas Value | Answer | LOINC |
|  | Prefer not to say | 0.9384738212377893 | 36311156 | Meas Value | Answer | LOINC |
|  | Decline to answer | 0.9287664901890188 | 36210418 | Meas Value | Answer | LOINC |
|  | Refused to answer | 0.9165109240083984 | 36032578 | Meas Value | Answer | LOINC |
|  | I choose not to answer this question | 0.9156258212013385 | 37079361 | Meas Value | Answer | LOINC |
|  | No - This question does not apply to me/I prefer not to answer | 0.9060141214522496 | 1621154 | Meas Value | Answer | LOINC |
|  | Unable to answer | 0.8989359464052384 | 45881125 | Meas Value | Answer | LOINC |
|  | Other/Unknown/Refuse To Answer | 0.8971773250061255 | 45877393 | Meas Value | Answer | LOINC |
|  | Unable to answer or no response | 0.8835975197540338 | 45885006 | Meas Value | Answer | LOINC |
|  | Don't know/refused | 0.8617725311007554 | 45876763 | Meas Value | Answer | LOINC |
| What was your sex assigned at birth? Other (specify) | Sex assigned at birth | 0.8796437441960989 | 46235213 | Observation | Clinical Observation | LOINC |
|  | Other (specify) | 0.8578062310352703 | 45883155 | Meas Value | Answer | LOINC |
|  | Sex assigned^at birth | 0.852286984 | 1023297 | Observation | LOINC Component | LOINC |
|  | Sex assigned | 0.8445320991541158 | 1013495 | Observation | LOINC Component | LOINC |
|  | Some Other Race (specify) | 0.8404416082293225 | 45882402 | Meas Value | Answer | LOINC |
|  | Sex of baby at delivery | 0.8398352138977829 | 4092760 | Observation | Observable Entity | SNOMED |
|  | Recorded sex or gender | 0.8393357507670831 | 1988369 | Observation | Clinical Observation | LOINC |
|  | Recorded sex or gender | 0.8393357507670831 | 1992214 | Observation | LOINC Component | LOINC |
|  | Your gender | 0.8371331709546251 | 45883294 | Meas Value | Answer | LOINC |
|  | Sex of baby | 0.8359285162711657 | 4149371 | Observation | Observable Entity | SNOMED |
| Sexual Orientation- Do you think of yourself as... | Sexual orientation | 0.8991592074005981 | 4283657 | Observation | Observable Entity | SNOMED |
|  | Sexual orientation | 0.8989083302472348 | 1013446 | Observation | LOINC Component | LOINC |
|  | Sexual orientation | 0.8989083302472348 | 46235214 | Observation | Clinical Observation | LOINC |
|  | Your sexual orientation | 0.8916851691536113 | 45877496 | Meas Value | Answer | LOINC |
|  | Sexual orientation confusion | 0.8875268211756993 | 35624208 | Observation | Clinical Finding | SNOMED |
|  | Undecided about sexual orientation | 0.8785698569369347 | 42689512 | Observation | Clinical Finding | SNOMED |
|  | Sexual orientation unknown | 0.8727166798834717 | 4260977 | Observation | Clinical Finding | SNOMED |
|  | Concern about sexual orientation | 0.8721582864450637 | 4085354 | Observation | Clinical Finding | SNOMED |
|  | Are you sexually active or would you like to be sexually active | 0.8566335899437082 | 1011042 | Observation | LOINC Component | LOINC |
|  | Bisexual - predominantly homosexual | 0.8565447042979908 | 4036081 | Observation | Clinical Finding | SNOMED |
| Straight or heterosexual | Heterosexual | 0.9279173854367059 | 4069091 | Observation | Clinical Finding | SNOMED |
|  | Heterosexual | 0.9278950060728077 | 36310681 | Meas Value | Answer | LOINC |
|  | Straight | 0.9039105475258338 | 4134759 | Observation | Qualifier Value | SNOMED |
|  | Heterosexual relationship | 0.8839833525157362 | 4043047 | Condition | Clinical Finding | SNOMED |
|  | Straight on | 0.8796616891193915 | 4134760 | Observation | Qualifier Value | SNOMED |
|  | Bisexual - predominantly heterosexual | 0.8771788403297872 | 4043048 | Observation | Clinical Finding | SNOMED |
|  | Straightforward | 0.8763281450131544 | 4295939 | Observation | Qualifier Value | SNOMED |
|  | Previously heterosexual transsexual | 0.8750477028264246 | 43021158 | Condition | Clinical Finding | SNOMED |
|  | Homosexual | 0.8712266881901435 | 36303203 | Meas Value | Answer | LOINC |
|  | Sexual orientation | 0.8700221603813884 | 4283657 | Observation | Observable Entity | SNOMED |
| Lesbian or gay | Homosexual | 0.8992166243489786 | 36303203 | Meas Value | Answer | LOINC |
|  | Female homosexual | 0.8958592870660712 | 4230492 | Observation | Clinical Finding | SNOMED |
|  | Bisexual | 0.8896635729744088 | 4170582 | Observation | Clinical Finding | SNOMED |
|  | Bisexual | 0.889386906 | 36307527 | Meas Value | Answer | LOINC |
|  | Male homosexual | 0.8870497322650006 | 4328364 | Observation | Clinical Finding | SNOMED |
|  | Lesbia | 0.8792128494958067 | 4308162 | Observation | Organism | SNOMED |
|  | Heterosexual | 0.8790373736658533 | 4069091 | Observation | Clinical Finding | SNOMED |
|  | Heterosexual | 0.8790127943965323 | 36310681 | Meas Value | Answer | LOINC |
|  | Homophobic | 0.8757402333409108 | 44804188 | Observation | Qualifier Value | SNOMED |
|  | Bisexual - predominantly homosexual | 0.870273467 | 4036081 | Observation | Clinical Finding | SNOMED |
| Bisexual | Bisexual | 0.9999999999999982 | 36307527 | Meas Value | Answer | LOINC |
|  | Bisexual | 0.9999950869355146 | 4170582 | Observation | Clinical Finding | SNOMED |
|  | Bisexual - predominantly heterosexual | 0.920978093 | 4043048 | Observation | Clinical Finding | SNOMED |
|  | Bisexual - predominantly homosexual | 0.9207062414246349 | 4036081 | Observation | Clinical Finding | SNOMED |
|  | Heterosexual | 0.9077492979873478 | 4069091 | Observation | Clinical Finding | SNOMED |
|  | Heterosexual | 0.9076975341462455 | 36310681 | Meas Value | Answer | LOINC |
|  | Previously bisexual transsexual | 0.9022860227720458 | 43021970 | Condition | Clinical Finding | SNOMED |
|  | Homosexual | 0.9002880090608549 | 36303203 | Meas Value | Answer | LOINC |
|  | Sexually attracted to male and female sex | 0.8875793452007597 | 35623014 | Observation | Clinical Finding | SNOMED |
|  | Intersexuality | 0.8834419932731913 | 4048723 | Condition | Clinical Finding | SNOMED |
| Queer | Homosexual | 0.9052405371621125 | 36303203 | Meas Value | Answer | LOINC |
|  | Bisexual | 0.8868474713850505 | 4170582 | Observation | Clinical Finding | SNOMED |
|  | Bisexual | 0.8865794708863949 | 36307527 | Meas Value | Answer | LOINC |
|  | Homophobic | 0.883707872 | 44804188 | Observation | Qualifier Value | SNOMED |
|  | Male homosexual | 0.8795475501181981 | 4328364 | Observation | Clinical Finding | SNOMED |
|  | Heterosexual | 0.8775645043374714 | 4069091 | Observation | Clinical Finding | SNOMED |
|  | Heterosexual | 0.8775589417166274 | 36310681 | Meas Value | Answer | LOINC |
|  | Female homosexual | 0.8770128012646728 | 4230492 | Observation | Clinical Finding | SNOMED |
|  | Transsexual | 0.8734967138783759 | 4231241 | Condition | Clinical Finding | SNOMED |
|  | Transsexual | 0.8731848009611947 | 45882199 | Meas Value | Answer | LOINC |
| Pansexual | Bisexual | 0.921031497 | 4170582 | Observation | Clinical Finding | SNOMED |
|  | Bisexual | 0.9208919582898152 | 36307527 | Meas Value | Answer | LOINC |
|  | Intersexuality | 0.8942666618211261 | 4048723 | Condition | Clinical Finding | SNOMED |
|  | Heterosexual | 0.8905142112163332 | 36310681 | Meas Value | Answer | LOINC |
|  | Heterosexual | 0.8905103947130628 | 4069091 | Observation | Clinical Finding | SNOMED |
|  | Homosexual | 0.878853105 | 36303203 | Meas Value | Answer | LOINC |
|  | Sexually attracted to male and female sex | 0.8756917220332929 | 35623014 | Observation | Clinical Finding | SNOMED |
|  | Intersex | 0.874642544 | 46273637 | Condition | Clinical Finding | SNOMED |
|  | Sexually attracted to neither male nor female sex | 0.8733099954994676 | 35623015 | Observation | Clinical Finding | SNOMED |
|  | Previously bisexual transsexual | 0.8715012531831271 | 43021970 | Condition | Clinical Finding | SNOMED |
| Questioning | Direct questioning | 0.9078365344455986 | 4233460 | Observation | Procedure | SNOMED |
|  | Repetitive questioning | 0.8937586324762592 | 43531646 | Condition | Clinical Finding | SNOMED |
|  | Ability to ask questions | 0.8856155679089481 | 4120926 | Observation | Observable Entity | SNOMED |
|  | Question | 0.8845595589357494 | 1012456 | Observation | LOINC System | LOINC |
|  | Does ask questions | 0.8803129227116981 | 4124949 | Condition | Clinical Finding | SNOMED |
|  | Difficulty asking questions | 0.8789104660658723 | 4127681 | Condition | Clinical Finding | SNOMED |
|  | Questionable | 0.8747054165612073 | 36308910 | Meas Value | Answer | LOINC |
|  | Suspicion | 0.8718270430020967 | 45768444 | Condition | Clinical Finding | SNOMED |
|  | Able to ask questions | 0.8685540340167431 | 4123718 | Condition | Clinical Finding | SNOMED |
|  | Thinking | 0.8674831873356135 | 4230784 | Observation | Observable Entity | SNOMED |
| Asexual | Sexually attracted to neither male nor female sex | 0.879833962 | 35623015 | Observation | Clinical Finding | SNOMED |
|  | Bisexual | 0.8782044168066522 | 4170582 | Observation | Clinical Finding | SNOMED |
|  | Bisexual | 0.8780468288870749 | 36307527 | Meas Value | Answer | LOINC |
|  | Asexual dwarfism | 0.8779875529724155 | 4326901 | Condition | Clinical Finding | SNOMED |
|  | Heterosexual | 0.877254635 | 4069091 | Observation | Clinical Finding | SNOMED |
|  | Heterosexual | 0.8772354461253051 | 36310681 | Meas Value | Answer | LOINC |
|  | Previously asexual transsexual | 0.8684380764445443 | 43021157 | Condition | Clinical Finding | SNOMED |
|  | Asocial behavior | 0.8653513740972202 | 4298668 | Condition | Clinical Finding | SNOMED |
|  | Sexually abstinent | 0.8640843949096513 | 764423 | Condition | Clinical Finding | SNOMED |
|  | Isosexual virilization | 0.859274804 | 4163684 | Condition | Clinical Finding | SNOMED |
| Something else | Something else | 0.9999999999999996 | 45882018 | Meas Value | Answer | LOINC |
|  | Other things | 0.9162376975703276 | 45876567 | Meas Value | Answer | LOINC |
|  | Other | 0.8970536119499477 | 9177 | Observation | Qualifier Value | SNOMED |
|  | Other | 0.8969392008865698 | 1032802 | Observation | LOINC Component | LOINC |
|  | Other | 0.8969392008865698 | 3040314 | Observation | Survey | LOINC |
|  | Other | 0.8969392008865698 | 21498861 | Meas Value | Answer | LOINC |
|  | Other | 0.8969392008865698 | 45878142 | Meas Value | Answer | LOINC |
|  | Somewhere else | 0.8933526595225842 | 45882581 | Meas Value | Answer | LOINC |
|  | Else | 0.8825629227623768 | 45879273 | Meas Value | Answer | LOINC |
|  | All other | 0.8725869442413988 | 36032719 | Meas Value | Answer | LOINC |
| Prefer not to answer | I prefer not to answer | 0.9719516354545338 | 1177221 | Meas Value | Answer | LOINC |
|  | Prefer not to say | 0.9381895648915678 | 36311156 | Meas Value | Answer | LOINC |
|  | Decline to answer | 0.9287401142086513 | 36210418 | Meas Value | Answer | LOINC |
|  | Refused to answer | 0.91647393 | 36032578 | Meas Value | Answer | LOINC |
|  | I choose not to answer this question | 0.9154184034592824 | 37079361 | Meas Value | Answer | LOINC |
|  | No - This question does not apply to me/I prefer not to answer | 0.9058590372376961 | 1621154 | Meas Value | Answer | LOINC |
|  | Unable to answer | 0.8985974577174314 | 45881125 | Meas Value | Answer | LOINC |
|  | Other/Unknown/Refuse To Answer | 0.8970932842712662 | 45877393 | Meas Value | Answer | LOINC |
|  | Unable to answer or no response | 0.8833741853812344 | 45885006 | Meas Value | Answer | LOINC |
|  | Don't know/refused | 0.861527047 | 45876763 | Meas Value | Answer | LOINC |
| Something else (specify) | Other (specify) | 0.9482354830425385 | 45883155 | Meas Value | Answer | LOINC |
|  | Other, specify | 0.9204035119298197 | 45883153 | Meas Value | Answer | LOINC |
|  | Something else | 0.9202776173404142 | 45882018 | Meas Value | Answer | LOINC |
|  | Some Other Race (specify) | 0.8891685723627194 | 45882402 | Meas Value | Answer | LOINC |
|  | Other (not listed) | 0.8831750170604732 | 45883627 | Meas Value | Answer | LOINC |
|  | Other: specify | 0.8810291131107831 | 1621139 | Meas Value | Answer | LOINC |
|  | Other | 0.8754273701217148 | 1032802 | Observation | LOINC Component | LOINC |
|  | Other | 0.8754273701217148 | 3040314 | Observation | Survey | LOINC |
|  | Other | 0.8754273701217148 | 21498861 | Meas Value | Answer | LOINC |
|  | Other | 0.8754273701217148 | 45878142 | Meas Value | Answer | LOINC |
| Are you currently pregnant? | Are you currently pregnant | 0.9731615588710373 | 1008103 | Observation | LOINC Component | LOINC |
|  | Have you ever been pregnant | 0.9081797174344876 | 1006219 | Observation | LOINC Component | LOINC |
|  | Currently pregnant | 0.8970328613326768 | 45884583 | Meas Value | Answer | LOINC |
|  | Are you and your partner currently trying to get pregnant | 0.8953209513940769 | 1006819 | Observation | LOINC Component | LOINC |
|  | Are you currently pregnant [PhenX] | 0.8939078672438016 | 40768856 | Observation | Clinical Observation | LOINC |
|  | Pregnant | 0.8743193574219796 | 4299535 | Condition | Clinical Finding | SNOMED |
|  | Pregnant | 0.873994865 | 45881770 | Meas Value | Answer | LOINC |
|  | Are you pregnant, or is there a chance you could become pregnant during the next month | 0.8724788471924271 | 1008292 | Observation | LOINC Component | LOINC |
|  | Were you trying to get pregnant | 0.8708407074465149 | 1018184 | Observation | LOINC Component | LOINC |
|  | Were you trying to get pregnant | 0.8708324470655293 | 1176388 | Observation | Clinical Observation | LOINC |
| What category best describes your current relationship status? | Interpersonal Relationship Status | 0.868998445 | 1028696 | Observation | LOINC Component | LOINC |
|  | Marital status | 0.8560276092996927 | 4053609 | Observation | Observable Entity | SNOMED |
|  | Marital status | 0.8557670058316833 | 1032587 | Observation | LOINC Component | LOINC |
|  | Marital status | 0.8557670058316833 | 3046344 | Observation | Clinical Observation | LOINC |
|  | In general, how would you rate your satisfaction with you social activities and relationships | 0.8545706889817033 | 1004598 | Observation | LOINC Component | LOINC |
|  | Discussion about relationship | 0.8428819586663068 | 44809761 | Procedure | Procedure | SNOMED |
|  | Social Contact Status | 0.8407055462810493 | 1028717 | Observation | LOINC Component | LOINC |
|  | Current living arrangement | 0.8404037804256926 | 1032153 | Observation | LOINC Component | LOINC |
|  | Current living arrangement | 0.8404037804256926 | 3051968 | Observation | Survey | LOINC |
|  | In general, how would you rate your satisfaction with you social activities and relationships [PROMIS] | 0.8385197447409908 | 40764342 | Observation | Survey | LOINC |
| Divorced | Divorced | 0.9999982345483419 | 45883375 | Meas Value | Answer | LOINC |
|  | Divorced | 0.9999829197578163 | 4069297 | Observation | Clinical Finding | SNOMED |
|  | Separated/Divorced | 0.9543299890189079 | 45879082 | Meas Value | Answer | LOINC |
|  | Separated or divorced | 0.9463860836629544 | 1620675 | Meas Value | Answer | LOINC |
|  | Divorce | 0.9251565799700081 | 4268603 | Observation | Clinical Finding | SNOMED |
|  | Married | 0.9189859029883801 | 4338692 | Observation | Clinical Finding | SNOMED |
|  | Married | 0.9188370539574344 | 45876756 | Meas Value | Answer | LOINC |
|  | Widowed | 0.9182446712132802 | 4143188 | Observation | Clinical Finding | SNOMED |
|  | Widowed | 0.9181423781395883 | 45883711 | Meas Value | Answer | LOINC |
|  | Remarried | 0.9102738557214398 | 4150598 | Observation | Clinical Finding | SNOMED |
| Married | Married | 0.9999979679344984 | 45876756 | Meas Value | Answer | LOINC |
|  | Married | 0.9999956640750112 | 4338692 | Observation | Clinical Finding | SNOMED |
|  | Remarried | 0.9359312952787677 | 4150598 | Observation | Clinical Finding | SNOMED |
|  | Divorced | 0.9188938163997434 | 4069297 | Observation | Clinical Finding | SNOMED |
|  | Divorced | 0.9188703244384485 | 45883375 | Meas Value | Answer | LOINC |
|  | Unmarried | 0.9170079095407333 | 21499178 | Meas Value | Answer | LOINC |
|  | Legally married | 0.9119010798558985 | 4278461 | Observation | Clinical Finding | SNOMED |
|  | Marriage | 0.9094446908030336 | 4052602 | Observation | Clinical Finding | SNOMED |
|  | Living as married | 0.907748 | 36308574 | Meas Value | Answer | LOINC |
|  | Widowed | 0.9066321882296392 | 4143188 | Observation | Clinical Finding | SNOMED |
| Never married | Never married | 1.0000000000000002 | 45881671 | Meas Value | Answer | LOINC |
|  | Single, never married | 0.9321875379783978 | 45885268 | Meas Value | Answer | LOINC |
|  | Single, never married | 0.9321394934061146 | 4053854 | Observation | Clinical Finding | SNOMED |
|  | Unmarried | 0.9269484067089834 | 21499178 | Meas Value | Answer | LOINC |
|  | Single; has never married or lived as married | 0.9211482147047346 | 45883239 | Meas Value | Answer | LOINC |
|  | Married | 0.8992360545517964 | 4338692 | Observation | Clinical Finding | SNOMED |
|  | Married | 0.899046182 | 45876756 | Meas Value | Answer | LOINC |
|  | Widowed | 0.887405085 | 4143188 | Observation | Clinical Finding | SNOMED |
|  | Widowed | 0.8872402034873831 | 45883711 | Meas Value | Answer | LOINC |
|  | Parents not married | 0.8856136822977413 | 21498925 | Meas Value | Answer | LOINC |
| Separated | Separated | 1.0000000000000029 | 45884459 | Meas Value | Answer | LOINC |
|  | Separated | 0.9972626618186544 | 4027529 | Observation | Clinical Finding | SNOMED |
|  | Separate | 0.9047110063758144 | 4139956 | Observation | Qualifier Value | SNOMED |
|  | With separation | 0.8971232683241309 | 4136122 | Observation | Qualifier Value | SNOMED |
|  | Separated/Divorced | 0.8961157555483209 | 45879082 | Meas Value | Answer | LOINC |
|  | Separated or divorced | 0.8922902308571894 | 1620675 | Meas Value | Answer | LOINC |
|  | Separation | 0.8853031490262321 | 4042921 | Observation | Morph Abnormality | SNOMED |
|  | Separation | 0.8853031490262321 | 4053101 | Observation | Clinical Finding | SNOMED |
|  | Disconnected | 0.8800359487174909 | 45882367 | Meas Value | Answer | LOINC |
|  | Division | 0.8799934425116289 | 4041107 | Procedure | Procedure | SNOMED |
| Widowed | Widowed | 1.0000000000000009 | 45883711 | Meas Value | Answer | LOINC |
|  | Widowed | 0.999995232 | 4143188 | Observation | Clinical Finding | SNOMED |
|  | Widower | 0.9661225355519486 | 4302155 | Observation | Clinical Finding | SNOMED |
|  | Widow | 0.9506183858418918 | 4149091 | Observation | Clinical Finding | SNOMED |
|  | Widow | 0.9505810404247349 | 45879694 | Meas Value | Answer | LOINC |
|  | Divorced | 0.9182079418099879 | 45883375 | Meas Value | Answer | LOINC |
|  | Divorced | 0.9180876714069334 | 4069297 | Observation | Clinical Finding | SNOMED |
|  | Remarried | 0.9072264353872839 | 4150598 | Observation | Clinical Finding | SNOMED |
|  | Married | 0.9065958238216459 | 4338692 | Observation | Clinical Finding | SNOMED |
|  | Married | 0.9064108015814323 | 45876756 | Meas Value | Answer | LOINC |
| Domestic partner | Domestic partner | 1.000000000000001 | 21499180 | Meas Value | Answer | LOINC |
|  | Domestic partner | 0.9999971844656541 | 4212893 | Observation | Social Context | SNOMED |
|  | Domestic partnership | 0.9710206417211208 | 4325710 | Observation | Clinical Finding | SNOMED |
|  | Domestic partner of subject | 0.9547828961620928 | 40485475 | Observation | Social Context | SNOMED |
|  | Domestic | 0.9032587239309028 | 4129943 | Observation | Location | SNOMED |
|  | Domestic partner abuse prevention | 0.9022198592913512 | 4303562 | Procedure | Procedure | SNOMED |
|  | Spouse/partner | 0.9012198175645874 | 36308102 | Meas Value | Answer | LOINC |
|  | Married/civil partner | 0.8956713623317486 | 44791567 | Observation | Clinical Finding | SNOMED |
|  | Unmarried partner | 0.8919031202372493 | 45883379 | Meas Value | Answer | LOINC |
|  | Living with partner | 0.8871787159929246 | 45883710 | Meas Value | Answer | LOINC |
| Prefer not to answer | I prefer not to answer | 0.9719876457389129 | 1177221 | Meas Value | Answer | LOINC |
|  | Prefer not to say | 0.9382847263902695 | 36311156 | Meas Value | Answer | LOINC |
|  | Decline to answer | 0.9287031242549465 | 36210418 | Meas Value | Answer | LOINC |
|  | Refused to answer | 0.91642402 | 36032578 | Meas Value | Answer | LOINC |
|  | I choose not to answer this question | 0.9154130555902008 | 37079361 | Meas Value | Answer | LOINC |
|  | No - This question does not apply to me/I prefer not to answer | 0.9058708138354414 | 1621154 | Meas Value | Answer | LOINC |
|  | Unable to answer | 0.8986322951148931 | 45881125 | Meas Value | Answer | LOINC |
|  | Other/Unknown/Refuse To Answer | 0.8970410725628549 | 45877393 | Meas Value | Answer | LOINC |
|  | Unable to answer or no response | 0.8833838251597624 | 45885006 | Meas Value | Answer | LOINC |
|  | Don't know/refused | 0.861506706 | 45876763 | Meas Value | Answer | LOINC |
| What is the highest grade or level of training/school you have completed or the highest degree you have received? | What is the highest grade or level of school you have completed or the highest degree you have received | 0.9552745090033141 | 1005889 | Observation | LOINC Component | LOINC |
|  | What is the highest grade or level of schooling you completed | 0.9317228748291919 | 1009992 | Observation | LOINC Component | LOINC |
|  | What is the highest grade or level of schooling you completed [SAMHSA] | 0.8983199535098322 | 40771091 | Observation | Survey | LOINC |
|  | What is the highest grade or level of school you have completed or the highest degree you have received [NHANES] | 0.8982628236384369 | 40766232 | Observation | Clinical Observation | LOINC |
|  | What is the highest level of school this patient has completed | 0.8750177850648383 | 1010095 | Observation | LOINC Component | LOINC |
|  | Highest level of education | 0.8725038051573102 | 1030755 | Observation | LOINC Component | LOINC |
|  | Highest level of education | 0.8725038051573102 | 42528763 | Observation | Clinical Observation | LOINC |
|  | Graduate level degree or coursework | 0.8628964565177348 | 45877716 | Meas Value | Answer | LOINC |
|  | What is the highest level of school this patient has completed [CARE] | 0.8571338664741138 | 40771893 | Observation | Survey | LOINC |
|  | Graduated from a college or university | 0.8530101304531221 | 45884846 | Meas Value | Answer | LOINC |
| Did not complete high school | Some high school, but did not graduate | 0.9404946617279789 | 1990281 | Meas Value | Answer | LOINC |
|  | More than eighth grade, but did not graduate from high school | 0.9264170371602742 | 45877233 | Meas Value | Answer | LOINC |
|  | Went to college, but did not graduate | 0.9227784577594867 | 45880186 | Meas Value | Answer | LOINC |
|  | No schooling completed | 0.9132813130284848 | 45877928 | Meas Value | Answer | LOINC |
|  | Less than high school degree | 0.8994862476196706 | 37079293 | Meas Value | Answer | LOINC |
|  | Went to a business, trade, or vocational school instead of high school | 0.8961187838734436 | 45884844 | Meas Value | Answer | LOINC |
|  | High school graduate | 0.8925964755768865 | 45884464 | Meas Value | Answer | LOINC |
|  | High school graduate or GED completed | 0.8891898976424641 | 45882125 | Meas Value | Answer | LOINC |
|  | 12th grade, no diploma | 0.88564711 | 45884461 | Meas Value | Answer | LOINC |
|  | 9th - 12th grade, no diploma | 0.8821551826001957 | 45880353 | Meas Value | Answer | LOINC |
| GED or equivalent | GED or equivalent | 0.9999912432611084 | 45881673 | Meas Value | Answer | LOINC |
|  | High school graduate or GED completed | 0.8851036373362697 | 45882125 | Meas Value | Answer | LOINC |
|  | High school graduate or GED | 0.8846331813406877 | 1990495 | Meas Value | Answer | LOINC |
|  | High school diploma or GED | 0.8780975697686557 | 37079292 | Meas Value | Answer | LOINC |
|  | PhD (or equivalent) | 0.865256334 | 21498578 | Meas Value | Answer | LOINC |
|  | Graduate level degree or coursework | 0.8639124777044912 | 45877716 | Meas Value | Answer | LOINC |
|  | Completed a GED | 0.8613519531477404 | 45883293 | Meas Value | Answer | LOINC |
|  | Educated to high school level | 0.8528929654775314 | 43021808 | Observation | Clinical Finding | SNOMED |
|  | Educated to senior high school level | 0.8516032580114844 | 43020395 | Observation | Clinical Finding | SNOMED |
|  | Graduate degree | 0.8494673458316356 | 45880652 | Meas Value | Answer | LOINC |
| High school diploma | High school degree | 0.9580988486850943 | 1619783 | Observation | LOINC Component | LOINC |
|  | High school diploma or GED | 0.9521505232489292 | 37079292 | Meas Value | Answer | LOINC |
|  | High school education | 0.930936917 | 1620880 | Meas Value | Answer | LOINC |
|  | High school graduate | 0.9298112856026937 | 45884464 | Meas Value | Answer | LOINC |
|  | High school graduate or GED | 0.9231431337389848 | 1990495 | Meas Value | Answer | LOINC |
|  | High school graduate or GED completed | 0.9161062948446176 | 45882125 | Meas Value | Answer | LOINC |
|  | Less than high school degree | 0.9103106900531148 | 37079293 | Meas Value | Answer | LOINC |
|  | 12th grade, no diploma | 0.9087635292929955 | 45884461 | Meas Value | Answer | LOINC |
|  | 9th - 12th grade, no diploma | 0.90679149 | 45880353 | Meas Value | Answer | LOINC |
|  | High school | 0.9049503342796843 | 45876261 | Meas Value | Answer | LOINC |
| Some college credit | Some college credit but no degree | 0.9354757081414352 | 45884154 | Meas Value | Answer | LOINC |
|  | Some college | 0.9131049952246248 | 45876260 | Meas Value | Answer | LOINC |
|  | Community college | 0.8866008970839798 | 4331152 | Observation | Location | SNOMED |
|  | Community college | 0.8864052218180355 | 1989705 | Meas Value | Answer | LOINC |
|  | Some college or 2-year degree | 0.8858544645732641 | 1990329 | Meas Value | Answer | LOINC |
|  | Some college, no degree | 0.8835528587895248 | 45882538 | Meas Value | Answer | LOINC |
|  | Received higher education college education | 0.8726443232664688 | 4072737 | Observation | Clinical Finding | SNOMED |
|  | School grants | 0.8720103834719357 | 4059033 | Observation | Qualifier Value | SNOMED |
|  | Bachelor's degree or some college | 0.8714872288242294 | 45881150 | Meas Value | Answer | LOINC |
|  | Technical college | 0.8687000577865638 | 4330461 | Observation | Location | SNOMED |
| 1 or more years of college credit | Some college or 2-year degree | 0.8936491031037661 | 1990329 | Meas Value | Answer | LOINC |
|  | Some college credit but no degree | 0.8903820043319036 | 45884154 | Meas Value | Answer | LOINC |
|  | Bachelor's degree or some college | 0.870203697 | 45881150 | Meas Value | Answer | LOINC |
|  | Years of education | 0.8693842834794352 | 1015298 | Observation | LOINC Component | LOINC |
|  | More than 4-yearcollege degree | 0.865333375 | 1990213 | Meas Value | Answer | LOINC |
|  | Some college | 0.8642857642852518 | 45876260 | Meas Value | Answer | LOINC |
|  | Enrolled in college or trade school, including four-year colleges, community colleges | 0.8578095969273095 | 1989028 | Observation | Survey | LOINC |
|  | Enrolled in college or trade school, including four-year colleges, community colleges | 0.8578095969273095 | 1992368 | Observation | LOINC Component | LOINC |
|  | Graduate level degree or coursework | 0.8576378641102207 | 45877716 | Meas Value | Answer | LOINC |
|  | Graduated from a college or university | 0.8544992712358271 | 45884846 | Meas Value | Answer | LOINC |
| Associate's Degree or Vocational Training Program (e.g. | Associate degree: occupational, technical, or vocational program | 0.9222191219091848 | 45881674 | Meas Value | Answer | LOINC |
|  | Associate degree (e.g., AA, AS) | 0.8866218523496806 | 45880354 | Meas Value | Answer | LOINC |
|  | Associate degree: academic program | 0.8808562154067939 | 45884462 | Meas Value | Answer | LOINC |
|  | Vocational training | 0.8745238591278358 | 4205153 | Procedure | Procedure | SNOMED |
|  | Vocational schooling | 0.8674828693261054 | 4259284 | Procedure | Procedure | SNOMED |
|  | Pre-vocational training | 0.8631538438733697 | 4043221 | Procedure | Procedure | SNOMED |
|  | Vocational training trainee | 0.8617464070033793 | 4009197 | Observation | Social Context | SNOMED |
|  | Vocational retraining | 0.8571737629482835 | 4338377 | Procedure | Procedure | SNOMED |
|  | Associate degree - Nursing | 0.8567688388748408 | 46237239 | Meas Value | Answer | LOINC |
|  | Some college or 2-year degree | 0.8554138677074797 | 1990329 | Meas Value | Answer | LOINC |
| Bachelor's Degree (e.g. | Bachelor's degree (e.g., BA, AB, BS) | 0.9386630835608821 | 45884155 | Meas Value | Answer | LOINC |
|  | Bachelor's degree | 0.9294041793857614 | 45876259 | Meas Value | Answer | LOINC |
|  | Bachelor's degree or some college | 0.8893630727850957 | 45881150 | Meas Value | Answer | LOINC |
|  | Master's degree (e.g., MA, MS, MEng, MEd, MSW, MBA) | 0.8889622546372425 | 45878298 | Meas Value | Answer | LOINC |
|  | Associate degree (e.g., AA, AS) | 0.8792824294462829 | 45880354 | Meas Value | Answer | LOINC |
|  | Baccalaureate degree | 0.8792637952649326 | 21499080 | Meas Value | Answer | LOINC |
|  | Doctoral degree (e.g., PhD, EdD) | 0.8777826492215267 | 37079105 | Meas Value | Answer | LOINC |
|  | University undergraduate degree | 0.8760908780845347 | 1620325 | Meas Value | Answer | LOINC |
|  | Bachelor | 0.874311563 | 4185851 | Observation | Clinical Finding | SNOMED |
|  | Doctorate (e.g., PhD, EdD) or Professional degree (e.g., MD, DDS, DVM, LLB, JD) | 0.8690334908692359 | 45878299 | Meas Value | Answer | LOINC |
| Graduate degree (MSW | Master's degree (e.g., MA, MS, MEng, MEd, MSW, MBA) | 0.9058572257968684 | 45878298 | Meas Value | Answer | LOINC |
|  | Graduate degree | 0.9033547639365063 | 45880652 | Meas Value | Answer | LOINC |
|  | Master's degree | 0.8963982285026711 | 21499259 | Meas Value | Answer | LOINC |
|  | Graduate level degree or coursework | 0.8777193031327872 | 45877716 | Meas Value | Answer | LOINC |
|  | Postgraduate degree | 0.8770380089560423 | 1620472 | Meas Value | Answer | LOINC |
|  | Doctoral degree | 0.873231711 | 21499304 | Meas Value | Answer | LOINC |
|  | Medical social worker | 0.8692869029831324 | 4010003 | Observation | Social Context | SNOMED |
|  | Graduate | 0.8677195075194885 | 4151179 | Observation | Social Context | SNOMED |
|  | Master's degree - Nursing | 0.8676713248676372 | 46237694 | Meas Value | Answer | LOINC |
|  | Social worker | 0.8636858881094212 | 4024166 | Observation | Social Context | SNOMED |
| Other | Other | 0.9999989198507514 | 9177 | Observation | Qualifier Value | SNOMED |
|  | Other | 0.9999893327846575 | 1032802 | Observation | LOINC Component | LOINC |
|  | Other | 0.9999893327846575 | 3040314 | Observation | Survey | LOINC |
|  | Other | 0.9999893327846575 | 21498861 | Meas Value | Answer | LOINC |
|  | Other | 0.9999893327846575 | 45878142 | Meas Value | Answer | LOINC |
|  | Others | 0.9606992063211545 | 40776267 | Observation | LOINC Component | LOINC |
|  | Others | 0.960612458 | 1620997 | Meas Value | Answer | LOINC |
|  | OTHER | 0.9324098928353518 | 8521 | Gender | Gender | Gender |
|  | All other | 0.9141604434452019 | 36032719 | Meas Value | Answer | LOINC |
|  | Else | 0.9090099105638773 | 45879273 | Meas Value | Answer | LOINC |
| What is your current employment status? | Employment status - current | 0.9277996748437886 | 1009468 | Observation | LOINC Component | LOINC |
|  | Employment status - current | 0.9277996748437886 | 40770471 | Observation | Clinical Observation | LOINC |
|  | Current occupational status | 0.9154372219383435 | 1009991 | Observation | LOINC Component | LOINC |
|  | Employment status | 0.9147230515455617 | 4073163 | Observation | Observable Entity | SNOMED |
|  | Employment status | 0.9144694399998923 | 1016241 | Observation | LOINC Component | LOINC |
|  | Current employment | 0.9047603211885517 | 1030349 | Observation | LOINC Component | LOINC |
|  | Employment report status | 0.8858518545159629 | 4150247 | Observation | Clinical Finding | SNOMED |
|  | Current employment - Reported | 0.8672778512968666 | 3012397 | Observation | Clinical Observation | LOINC |
|  | Employment | 0.8669884242063868 | 1989892 | Meas Value | Answer | LOINC |
|  | Employment | 0.8669175911915683 | 44804285 | Observation | Qualifier Value | SNOMED |
| Full-time employment | Full-time employment | 0.999999273 | 4053118 | Observation | Clinical Finding | SNOMED |
|  | Full-time work | 0.9708446681532069 | 37079092 | Meas Value | Answer | LOINC |
|  | Full-time | 0.9538444133095613 | 45880423 | Meas Value | Answer | LOINC |
|  | Employed full time | 0.9433485238429972 | 45885105 | Meas Value | Answer | LOINC |
|  | Working full time | 0.9391514402798021 | 1621071 | Meas Value | Answer | LOINC |
|  | Part-time employment | 0.9269115486702395 | 4059634 | Observation | Clinical Finding | SNOMED |
|  | Full time student | 0.9120730334282456 | 1620565 | Meas Value | Answer | LOINC |
|  | Full-time (Year-Round) | 0.9108903452622258 | 45883408 | Meas Value | Answer | LOINC |
|  | Employed part time | 0.9077775906172182 | 45879123 | Meas Value | Answer | LOINC |
|  | Full-time (Seasonal) | 0.9032936210440936 | 45882577 | Meas Value | Answer | LOINC |
| Not employed | Unemployed | 0.9380014165463326 | 4251171 | Observation | Clinical Finding | SNOMED |
|  | Unemployed | 0.9378517141517001 | 1002718 | Observation | LOINC Component | LOINC |
|  | Unemployed | 0.9378517141517001 | 45877709 | Meas Value | Answer | LOINC |
|  | Employed | 0.9281138558493851 | 4076340 | Observation | Clinical Finding | SNOMED |
|  | Employed | 0.9280254183229703 | 1988444 | Observation | Clinical Observation | LOINC |
|  | Employed | 0.9280254183229703 | 1991754 | Observation | LOINC Component | LOINC |
|  | Employed | 0.9280254183229703 | 45877708 | Meas Value | Answer | LOINC |
|  | Not employed at onset | 0.9213137987218497 | 45882357 | Meas Value | Answer | LOINC |
|  | Not seeking work | 0.9134578448154296 | 4074928 | Observation | Clinical Finding | SNOMED |
|  | Unemployed, not looking | 0.9104739402638139 | 1620906 | Meas Value | Answer | LOINC |
| Part-time employment | Part-time employment | 0.9999990333809624 | 4059634 | Observation | Clinical Finding | SNOMED |
|  | Part-time or temporary work | 0.9604847750884691 | 37079331 | Meas Value | Answer | LOINC |
|  | Part-time | 0.9570259424623213 | 45880422 | Meas Value | Answer | LOINC |
|  | Working part time | 0.9502729070063505 | 1620635 | Meas Value | Answer | LOINC |
|  | Employed part time | 0.9492497037205819 | 45879123 | Meas Value | Answer | LOINC |
|  | Full-time employment | 0.9269646732044452 | 4053118 | Observation | Clinical Finding | SNOMED |
|  | Part time student | 0.9247951808052188 | 1620715 | Meas Value | Answer | LOINC |
|  | Student in part-time education | 0.917509251 | 764654 | Observation | Social Context | SNOMED |
|  | Part-time (Seasonal) | 0.9172232088678565 | 45876792 | Meas Value | Answer | LOINC |
|  | Casual employment | 0.9143887247455983 | 44803988 | Observation | Qualifier Value | SNOMED |
| Have you ever applied for, or received, disability insurance for your pain condition? | Patient reported disability | 0.8585941589472764 | 44809160 | Condition | Clinical Finding | SNOMED |
|  | Have you ever seen a doctor about this pain | 0.8546316388128378 | 1008885 | Observation | LOINC Component | LOINC |
|  | Did you receive treatment | 0.853878371 | 1006857 | Observation | LOINC Component | LOINC |
|  | Has your child ever been diagnosed with a physical disability | 0.851025937 | 1007278 | Observation | LOINC Component | LOINC |
|  | Have you had medical problems as a result of your drug use | 0.8464436097100112 | 1010020 | Observation | LOINC Component | LOINC |
|  | Have you been diagnosed with any type of autoimmune disease - lupus, scleroderma, etc | 0.8456896516276274 | 1008387 | Observation | LOINC Component | LOINC |
|  | Have you ever been treated for your glaucoma | 0.8454254651445321 | 1006995 | Observation | LOINC Component | LOINC |
|  | Receiving disability living allowance | 0.8453411946870267 | 44805927 | Observation | Clinical Finding | SNOMED |
|  | Have you ever been diagnosed by a physician or other health professional as suffering from | 0.845264974 | 1007479 | Observation | LOINC Component | LOINC |
|  | Have you been treated with chemotherapy or other medication for this condition | 0.8440417235851309 | 1009170 | Observation | LOINC Component | LOINC |
| Are you currently attending school (including vocational or trade school)? | Vocational schooling | 0.8841082763541445 | 4259284 | Procedure | Procedure | SNOMED |
|  | Went to a business, trade, or vocational school after high school | 0.8737078764053245 | 45884845 | Meas Value | Answer | LOINC |
|  | Attending school | 0.8725885188342991 | 4072738 | Observation | Clinical Finding | SNOMED |
|  | Went to a business, trade, or vocational school instead of high school | 0.8643325381257722 | 45884844 | Meas Value | Answer | LOINC |
|  | Technical or trade school | 0.8633438116625746 | 45881537 | Meas Value | Answer | LOINC |
|  | Student in full time education | 0.8619033361208929 | 4137058 | Observation | Social Context | SNOMED |
|  | Enrolled in college or trade school, including four-year colleges, community colleges | 0.8611805632552864 | 1989028 | Observation | Survey | LOINC |
|  | Enrolled in college or trade school, including four-year colleges, community colleges | 0.8611805632552864 | 1992368 | Observation | LOINC Component | LOINC |
|  | School attendance | 0.8571051808323675 | 4027309 | Observation | Observable Entity | SNOMED |
|  | Vocational training | 0.8565009824347186 | 4205153 | Procedure | Procedure | SNOMED |
| Are you attending full time? | Full time student | 0.9021271093247005 | 1620565 | Meas Value | Answer | LOINC |
|  | Working full time | 0.8908676669219144 | 1621071 | Meas Value | Answer | LOINC |
|  | Student in full time education | 0.8864791214787882 | 4137058 | Observation | Social Context | SNOMED |
|  | Full-time | 0.8846335880715888 | 45880423 | Meas Value | Answer | LOINC |
|  | Was this a full-time or part-time job | 0.8769156597835386 | 1006089 | Observation | LOINC Component | LOINC |
|  | Employed full time | 0.8736541727361822 | 45885105 | Meas Value | Answer | LOINC |
|  | Full-time employment | 0.8733399390866744 | 4053118 | Observation | Clinical Finding | SNOMED |
|  | Full-time work | 0.8723925450266353 | 37079092 | Meas Value | Answer | LOINC |
|  | Part time student | 0.867389631 | 1620715 | Meas Value | Answer | LOINC |
|  | Have you ever worked full time, 30 hours per week or more, for 6 months or more | 0.863906159 | 1006443 | Observation | LOINC Component | LOINC |
| What is your address and zip code? | Street address | 0.8825645468190906 | 4160017 | Observation | Observable Entity | SNOMED |
|  | Current zip code | 0.8802608922250015 | 1005888 | Observation | LOINC Component | LOINC |
|  | Zip code unknown | 0.8683027965505118 | 1033455 | Observation | LOINC Component | LOINC |
|  | Zip code unknown | 0.8683027965505118 | 40757186 | Observation | Survey | LOINC |
|  | Organization ZIP code | 0.8682430417411389 | 46235219 | Observation | Clinical Observation | LOINC |
|  | Zip - post code | 0.8658153567662786 | 1007718 | Observation | LOINC Component | LOINC |
|  | Mailing address | 0.8624191326976974 | 1002606 | Observation | LOINC Component | LOINC |
|  | Postal code | 0.8610182890740721 | 1033454 | Observation | LOINC Component | LOINC |
|  | Zip or P.O. Box | 0.8553557124150363 | 45880084 | Meas Value | Answer | LOINC |
|  | Residence Zip +4 | 0.8522752682858238 | 45880085 | Meas Value | Answer | LOINC |

The demographics common data elements concepts used in testing this AI tool are publicly available from the NIH HEAL Common Data Elements (CDE) (heal.nih.gov) to allow broad reusability and alignment with existing work. The demographic concepts presented in this supplemental file were developed by the NIH HEAL Initiative Common Data Elements (CDE) program and were adapted to have additional answer choice subconcepts by the CDE Working Group of the IMPOWR (Integrative Management of chronic Pain and OUD for Whole Recovery) Network in 2021-2022. The CDE Working Group included representatives from the IMPOWR Coordination and Dissemination Center at Wake Forest University Health Sciences (R24DA055306, PI: Adams) and Research Centers at the University of Pittsburgh (RM1DA055311), Albert Einstein College of Medicine (RM1DA055437), Yale University (RM1DA055310), and University of New Mexico (RM1DA055301). Development of these variables included input from IMPOWR community partners to ensure relevance and cultural appropriateness. These variables were implemented in REDCap by the IMPOWR Coordination and Dissemination Center as part of the IMPOWR Network's data harmonization efforts and no clinical data from these centers were used in the concept mapping.

The IMPOWR Network is funded by the National Institute on Drug Abuse (NIDA) of the National Institutes of Health (NIH) through the NIH HEAL Initiative. The content is solely the responsibility of the authors and does not necessarily represent the official views of the National Institutes of Health.
