# Supplementary material for: Breaking Digital Health Barriers Through a Large Language Model–Based Tool for Automated Observational Medical Outcomes Partnership Mapping: Development and Validation Study
Source: J Med Internet Res. 2025 May 15;27:e69004. doi: 10.2196/69004 (PMC12123247; doi:10.2196/69004)
Supplement: Multimedia Appendix 2 [file jmir_v27i1e69004_app2.zip › IMPOWR-R24-REDCap-to-OMOP-main/docs/65_REDCap2OMOP.pdf]

REDCap2OMOP: A platform for ETLing REDCap projects into the OMOP CDM

Michael J. Gurley, Jeremy Warner, Yulia Bushmanova, Firas Wehbe

Background

REDCap[1] is a flexible web-based data capture system enabling users to define projects consisting of collections of variables named "instruments". REDCap is widely used in the healthcare informatics community for clinical trials and longitudinal research registries. REDCap's underlying entity-attribute-value (EAV) data structure enables elastic functionality. REDCap does not have a predefined structure or semantics. OMOP is a strictly defined common data model (CDM) that pre-specifies the structure and semantics of healthcare data. OMOP uses a relational data model to normalize the structure of clinical events (e.g., people, providers, visits, drug exposures, procedures, conditions and measurements). OMOP uses an ontology-based conceptual structure to normalize the semantics of clinical events. OMOP houses numerous widely used healthcare standard vocabularies in a uniform set of vocabulary tables. Each OMOP clinical event table draws from a domain of "standard" concepts that define the semantics of the clinical event. The OMOP vocabulary tables also contains non-standard source vocabularies to facilitate the transformation of source data into the OMOP CDM.

The COVID-19 & Cancer Consortium (CCC19) [2] is a multi-institutional longitudinal research registry that is collecting reports on adult patients with a current or historical invasive solid or hematologic malignancy who have been diagnosed with COVID-19. CCC19 uses a centralized REDCap project to allow multiple institutions to enter granular, uniformly organized information about the diagnosis, treatment and outcomes of COVID-19 and cancer diagnoses. The CCC19 project has a use case to convert its REDCap data to OMOP to enable the use of OHDSI's readymade suite of analytic tools and methods libraries. The motivation to perform this transformation is allow for the easier comparison of the REDCap data assets to non-REDCap data assets. To that end, CCC19 is developing an open source MIT-licensed platform, REDCap2OMOP[3], to handle the conversion of REDcap data to the OMOP CDM. REDCap2OMOP is being developed in generalized manner to allow it to apply to other REDCap projects.

Methods

The REDCap2OMOP platform consists of two primary components; (1) a browser-based interface (**Curator**) for managing REDCap data dictionary versions, OMOP vocabulary mappings and time point designations; and (2) ETL code (**Converter**) that applies these mappings and designations to a REDCap data export to populate an OMOP 5.3.1 instance.

Results

The CCC19 REDCap data dictionary is committed to the CCC19 Github repository. The Curator application is deployed on a publicly accessible HTTPS application server. The Curator application periodically pulls the CCC19 REDCap data dictionary from the CCC19 Github repository, calculates the delta to the latest curated REDCap data dictionary version, generates a new version if necessary and migrates prior curated OMOP vocabulary mappings and time point designations to the latest version. New REDCap variables and variable choices are marked as needing curation. To facilitate the selection of appropriate OMOP concepts for the curation process, the Curator database contains a full copy of the 5.3.1 OMOP vocabulary tables populated with the latest OMOP vocabulary release from Athena.

The Curator application is a password-protected Ruby on Rails 6.x application requiring authentication. A limited population of curators has accounts provisioned to allow for curation of CCC19 REDCap data

dictionary versions. The Curator interface supports the following curation tasks for a REDCap data dictionary version:

- Define if a REDCap variable should be mapped to an OMOP clinical event, person or person.
- Define if a REDCap variable choice should be mapped to an OMOP clinical event.
- Define the calculation of timepoints based on the interval calculation between REDCap variables.
- Designate REDCap variable/variable choices to time points.

Curator is a Ruby on Rails 6.x application that uses the Stimulus JavaScript framework backed by a PostgreSQL 13 database server. The Curator application allows for its centrally curated REDCap data dictionaries to be accessible via a RESTful API secured by OAuth2.

The REDCap2OMOP Converter code is a Ruby on Rails 6.x application backed by a PostgreSQL 13 database server deployable within a Docker image. The Converter code contains a fully compiled 5.3.1 OMOP database schema and a Rake task for loading the latest OMOP vocabulary release from Athena into the OMOP vocabulary tables. Splitting the Curator code from the Converter code will allow for the central management of REDCap Data Dictionary versions and OMOP vocabulary mappings and time point designations. Conversely, the Converter code can be deployable at multiple institutional locations: the central CCC19 institution or at satellite institutions that either have a local CCC19 REDCap project instantiated or have had their institutional data exported from the central CCC19 REDCap project.

Each deployed Converter instance will be granted OAuth 2 credentials to allow for importing the curated REDCap data dictionary versions from Curator's RESTful API. Converter is a password-protected Ruby 6.x on Rails application requiring authentication. A limited population of administrators have accounts provisioned to allow for execution of an ETL. Converter has a simple user interface allowing an administrator to choose a REDCap data dictionary version, ingest a REDCap project's full data via the RDCap API and initiate an ETL execution. The ETL execution will occur asynchronously in a background job. Converter validates that the REDCap full data export complies with the chosen REDCap data dictionary version. If the uploaded REDCap full data export is not in compliance, the upload is rejected and the compliance violations are reported to the user. If the uploaded REDCap full data export complies, the ETL perform a truncate and load ETL to the 5.3.1 OMOP instance, using the curated OMOP vocabulary mappings and time point designations. The results of the ETL execution are displayed to the user. All unmapped REDCap variables and REDCap variable choices will be displayed for inspection.

Conclusion

The REDCap2OMOP platform provides for a robust solution to the challenge of managing the ETL of evolving REDCap projects across newly published version of REDCap data dictionaries to the OMOP CDM.

References/Citations

1. REDCap Research Electronic Data Capture https://www.project-redcap.org/
2. The COVID-19 and Cancer Consortium: https://ccc19.org/
3. REDCap2OMOP: https://github.com/NUARIG/redcap2omop
