# Supplementary material for: Breaking Digital Health Barriers Through a Large Language Model–Based Tool for Automated Observational Medical Outcomes Partnership Mapping: Development and Validation Study
Source: J Med Internet Res. 2025 May 15;27:e69004. doi: 10.2196/69004 (PMC12123247; doi:10.2196/69004)
Supplement: Multimedia Appendix 2 [file jmir_v27i1e69004_app2.zip › IMPOWR-R24-REDCap-to-OMOP-main/docs/65_poster-REDCap2OMOP.pdf]

REDCap2OMOP:
A platform for ETLing REDCap projects into the OMOP CDM

PRESENTER: **Michael Gurley**

INTRO:

- Many REDCap proejcts want to convert their data to the OMOP CDM to make their REDCap[1] data comparable to other data assets and use OHDSI's readymade suite of analytic tools and methods libraries.
- The CCC19[2] registry has developed an open source MIT-licensed platform, REDCap2OMOP[3], to handle the conversion of REDcap data to the OMOP CDM.

METHODS

The REDCap2OMOP platform consists of two primary components;

1. A browser-based interface (**Curator**) for managing REDCap data dictionary versions, OMOP vocabulary mappings and time point designations.
2. ETL code (**Converter**) that applies these mappings and designations to a REDCap data export to populate an OMOP 5.3.1 instance.

RESULTS

- **Curator** ingests data dictionary versions, computes a delta, creates a new version if necessary, migrates prior mappings and supports the curation of new items in a user interface.
- **Convertor** pulls the curated mappings from **Curator** via a RESTful API, imports REDCap data from a REDCap project via the REDCap API and uses the mappings to ETL into an OMOP 5.3.1 instance.

Footnotes

1. REDCap Research Electronic Data Capture https://www.project-redcap.org/
2. The COVID-19 and Cancer Consortium: https://ccc19.org/
3. REDCap2OMOP: https://github.com/NUARIG/redcap2omop

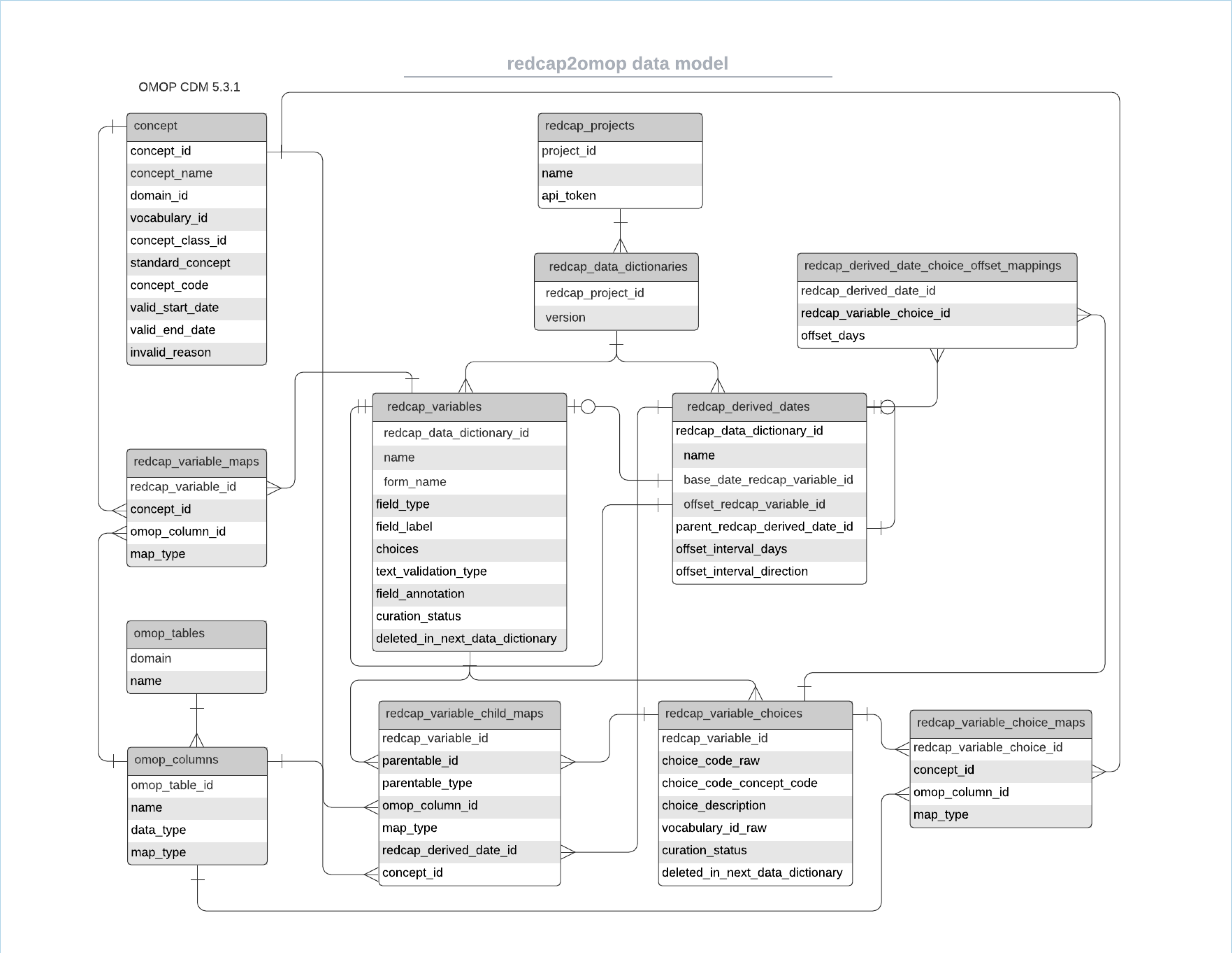

Take a picture to download the full paper

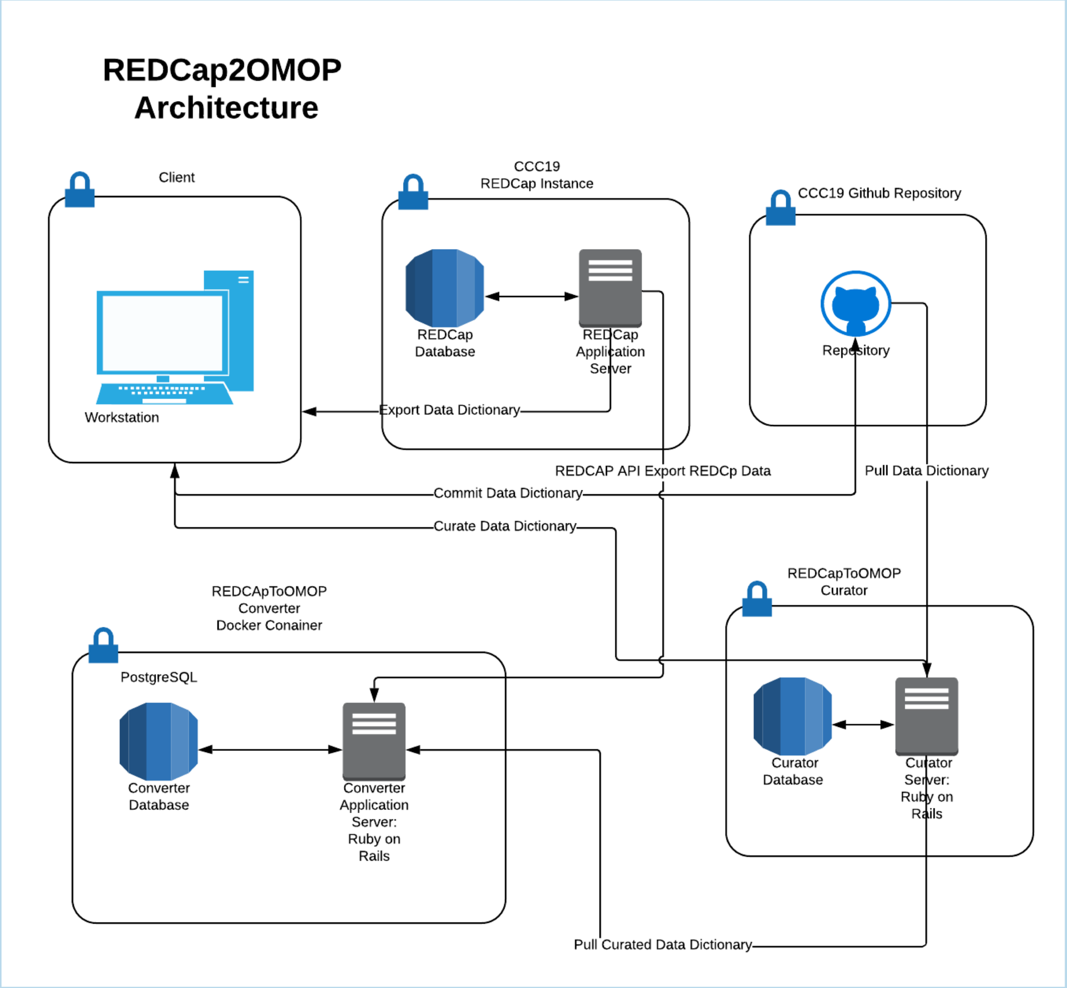

ETL Logic

- People, Providers and Death are handled separately. Must have enough variables to create people.
- Clinical Domain entities built from REDCap Variable or REDCap variable choices mapped to standard concepts. Subsidiary columns handled by child maps.
- CCC19 REDCap project does not support dates. Derived date logic was added.
- REDCap Variable map types:
  - OMOP Column
  - OMOP concept
  - OMOP concept choice
- REDCap Variable Choice map types:
  - OMOP concept

Take away

The REDCap2OMOP platform provides for a robust solution to the challenge of managing the ETL of evolving REDCap projects across newly published versions of REDCap data dictionaries to the OMOP CDM.

Jeremy Warner
Yulia Bushmanova
Firas Wehbe
