# Supplementary material for: Breaking Digital Health Barriers Through a Large Language Model–Based Tool for Automated Observational Medical Outcomes Partnership Mapping: Development and Validation Study
Source: J Med Internet Res. 2025 May 15;27:e69004. doi: 10.2196/69004 (PMC12123247; doi:10.2196/69004)
Supplement: Multimedia Appendix 2 [file jmir_v27i1e69004_app2.zip › IMPOWR-R24-REDCap-to-OMOP-main/frontend/public/index.html]

CDE To OMOP

You need to enable JavaScript to run this app.
