# Supplementary figures and images for: Breaking Digital Health Barriers Through a Large Language Model–Based Tool for Automated Observational Medical Outcomes Partnership Mapping: Development and Validation Study
Source: J Med Internet Res. 2025 May 15;27:e69004. doi: 10.2196/69004 (PMC12123247; doi:10.2196/69004)

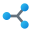

Supplement: Multimedia Appendix 2 [file jmir_v27i1e69004_app2.zip › IMPOWR-R24-REDCap-to-OMOP-main/frontend/public/icons8-connect-windows-11-color-32.png]

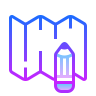

Supplement: Multimedia Appendix 2 [file jmir_v27i1e69004_app2.zip › IMPOWR-R24-REDCap-to-OMOP-main/frontend/public/logo192.png]

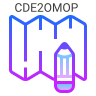

Supplement: Multimedia Appendix 2 [file jmir_v27i1e69004_app2.zip › IMPOWR-R24-REDCap-to-OMOP-main/frontend/public/logo1922.png]

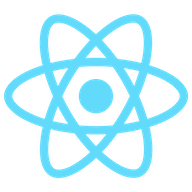

Supplement: Multimedia Appendix 2 [file jmir_v27i1e69004_app2.zip › IMPOWR-R24-REDCap-to-OMOP-main/frontend/public/logo192_2.png]

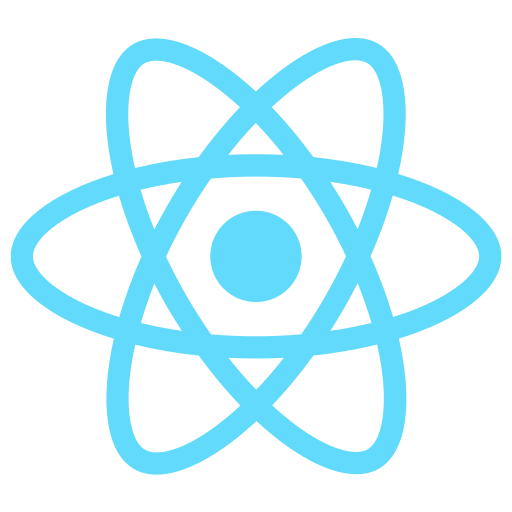

Supplement: Multimedia Appendix 2 [file jmir_v27i1e69004_app2.zip › IMPOWR-R24-REDCap-to-OMOP-main/frontend/public/logo512.png]

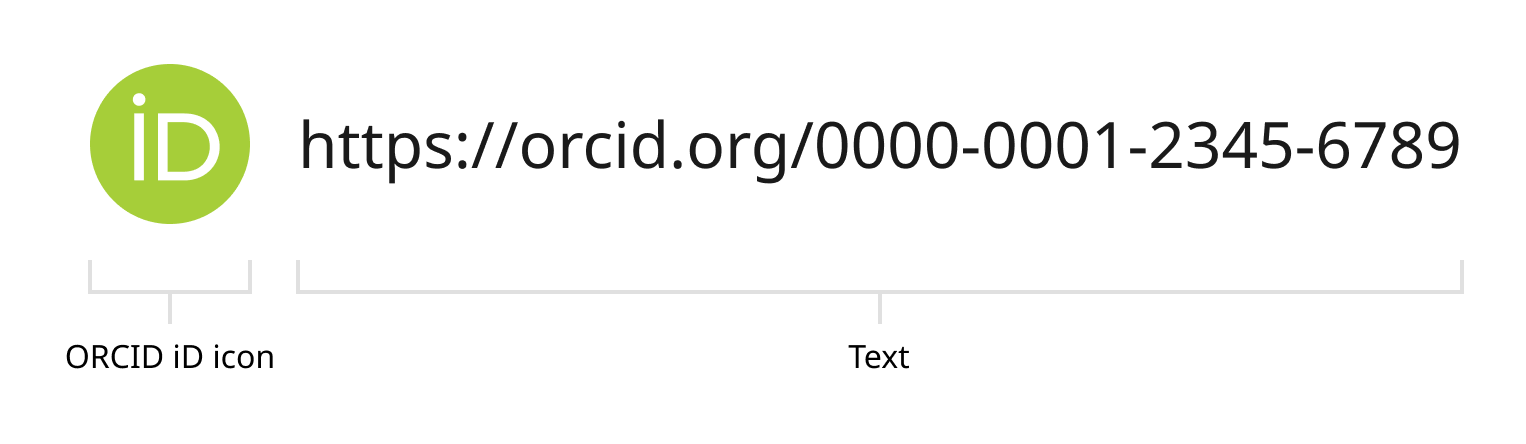

Supplement: Multimedia Appendix 2 [file jmir_v27i1e69004_app2.zip › IMPOWR-R24-REDCap-to-OMOP-main/frontend/src/assets/OrcidLogo.png]

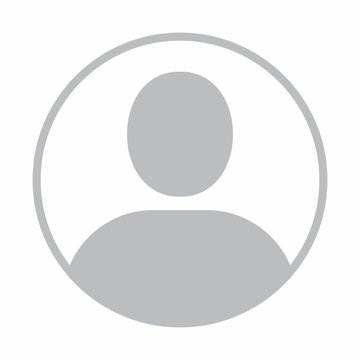

Supplement: Multimedia Appendix 2 [file jmir_v27i1e69004_app2.zip › IMPOWR-R24-REDCap-to-OMOP-main/frontend/src/assets/blank_avatar.jpg]

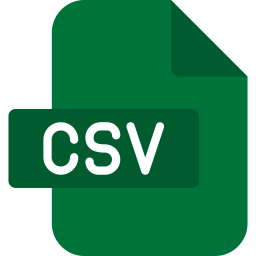

Supplement: Multimedia Appendix 2 [file jmir_v27i1e69004_app2.zip › IMPOWR-R24-REDCap-to-OMOP-main/frontend/src/assets/csv.png]

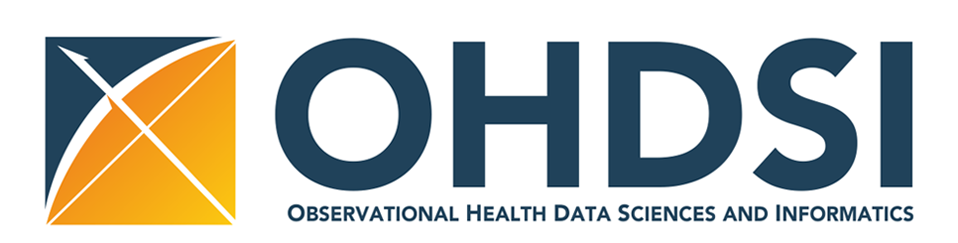

Supplement: Multimedia Appendix 2 [file jmir_v27i1e69004_app2.zip › IMPOWR-R24-REDCap-to-OMOP-main/frontend/src/assets/h243-ohdsi-logo-with-text.png]

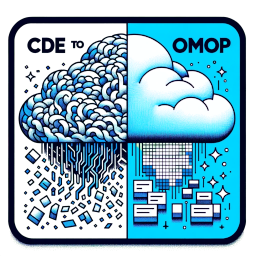

Supplement: Multimedia Appendix 2 [file jmir_v27i1e69004_app2.zip › IMPOWR-R24-REDCap-to-OMOP-main/frontend/src/assets/logo-old.png]

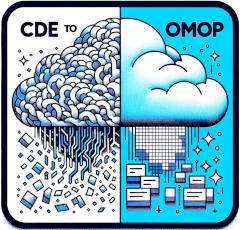

Supplement: Multimedia Appendix 2 [file jmir_v27i1e69004_app2.zip › IMPOWR-R24-REDCap-to-OMOP-main/frontend/src/assets/logo.png]

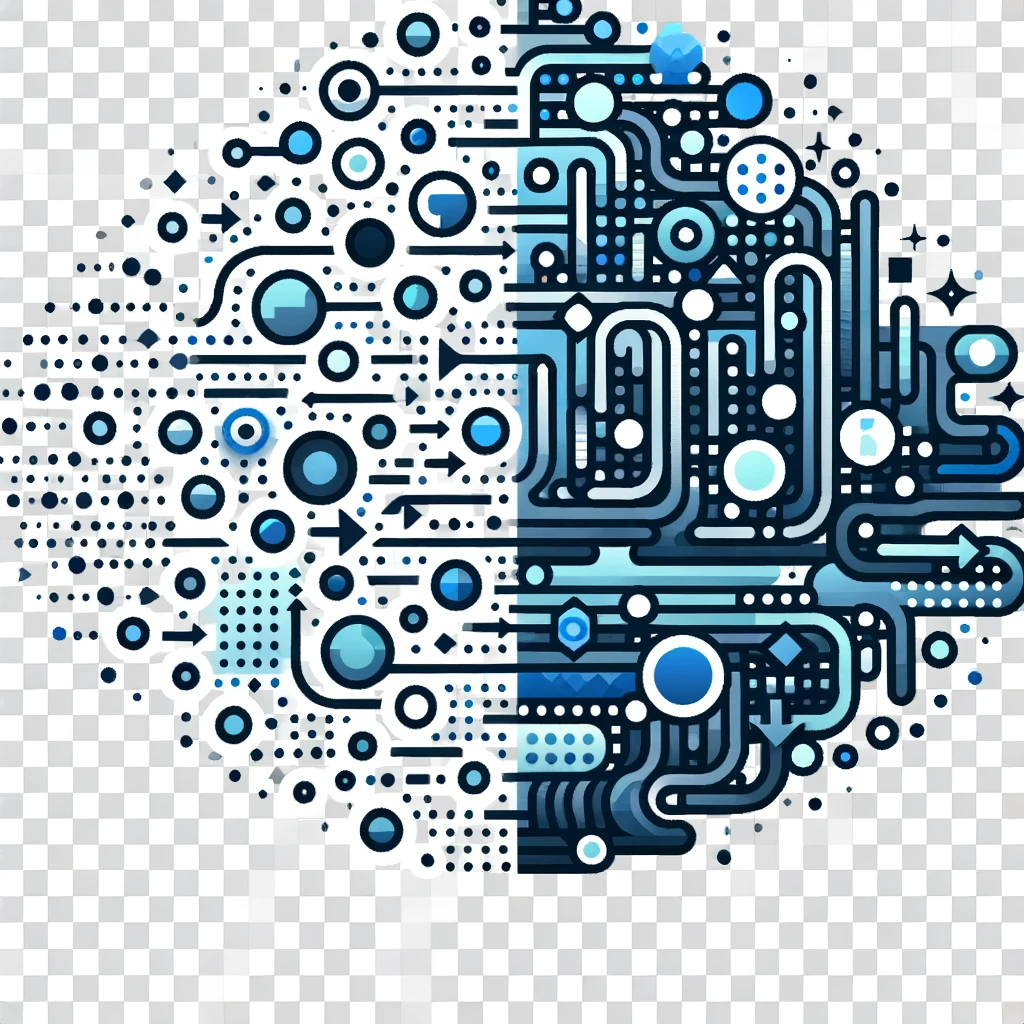

Supplement: Multimedia Appendix 2 [file jmir_v27i1e69004_app2.zip › IMPOWR-R24-REDCap-to-OMOP-main/frontend/src/assets/logo1.png]

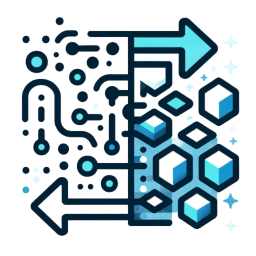

Supplement: Multimedia Appendix 2 [file jmir_v27i1e69004_app2.zip › IMPOWR-R24-REDCap-to-OMOP-main/frontend/src/assets/logo422.png]

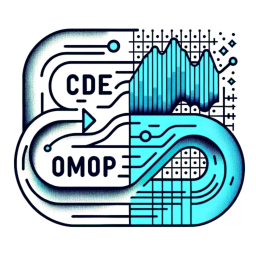

Supplement: Multimedia Appendix 2 [file jmir_v27i1e69004_app2.zip › IMPOWR-R24-REDCap-to-OMOP-main/frontend/src/assets/logo53.png]

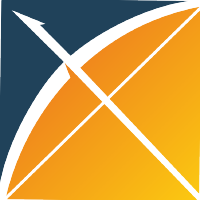

Supplement: Multimedia Appendix 2 [file jmir_v27i1e69004_app2.zip › IMPOWR-R24-REDCap-to-OMOP-main/frontend/src/assets/ohdsi_logo.png]

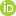

Supplement: Multimedia Appendix 2 [file jmir_v27i1e69004_app2.zip › IMPOWR-R24-REDCap-to-OMOP-main/frontend/src/assets/orcid_16x16.gif]

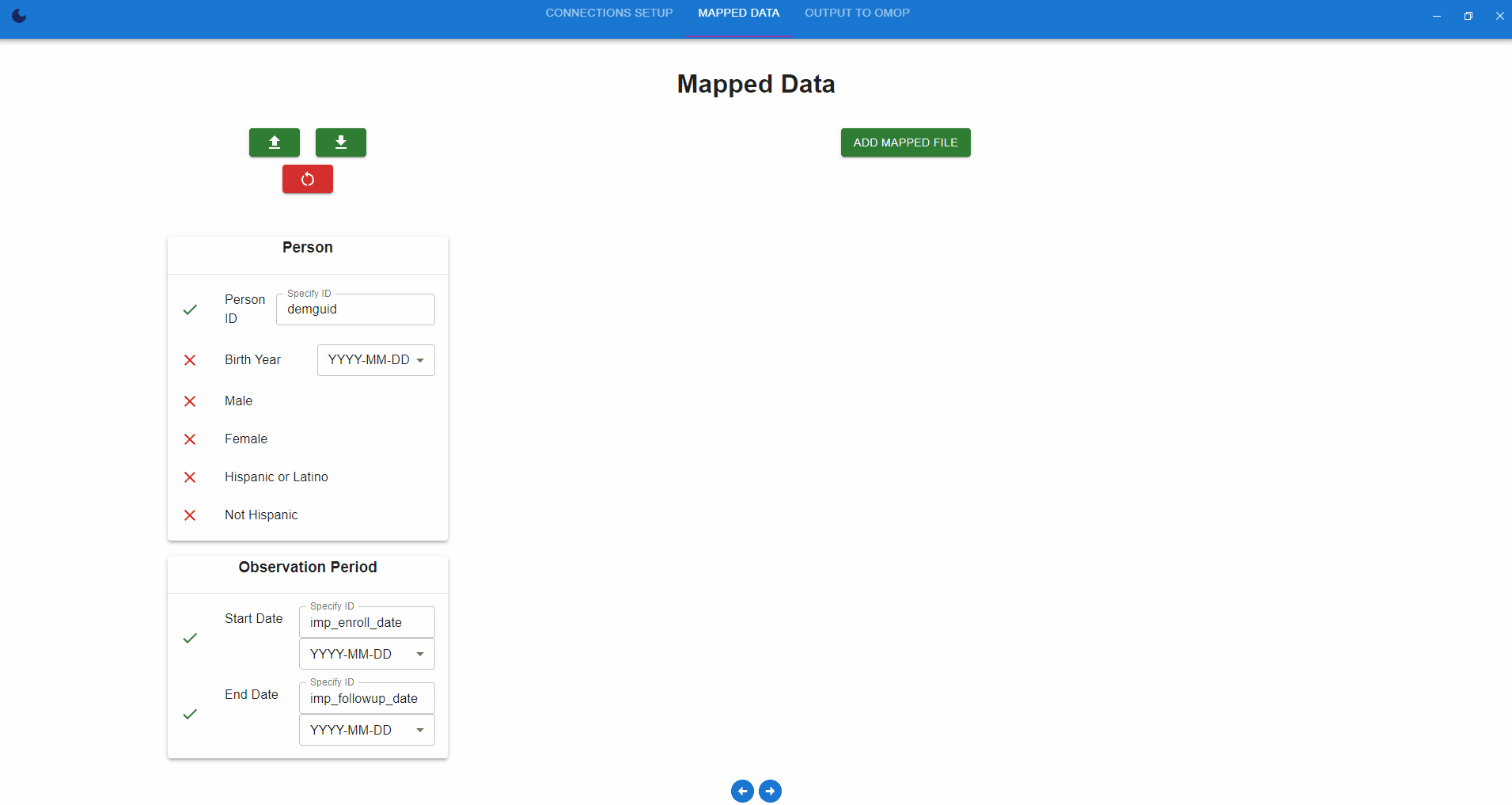

Supplement: Multimedia Appendix 2 [file jmir_v27i1e69004_app2.zip › IMPOWR-R24-REDCap-to-OMOP-main/frontend/src/assets/readme/desktopapp_add_file.gif]

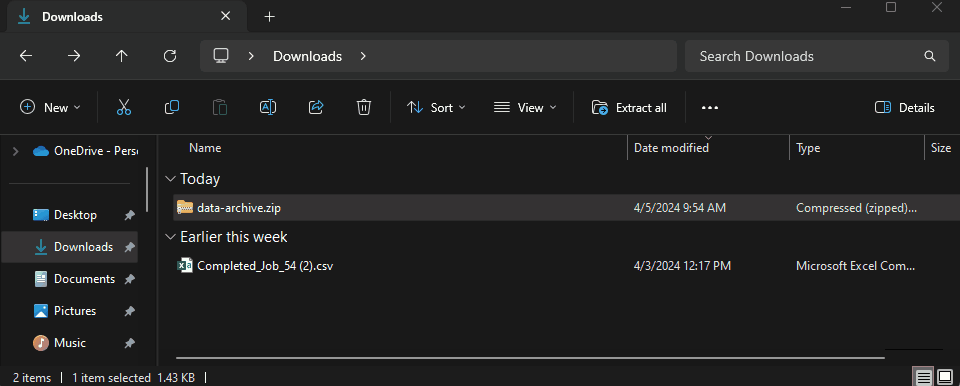

Supplement: Multimedia Appendix 2 [file jmir_v27i1e69004_app2.zip › IMPOWR-R24-REDCap-to-OMOP-main/frontend/src/assets/readme/desktopapp_files.gif]

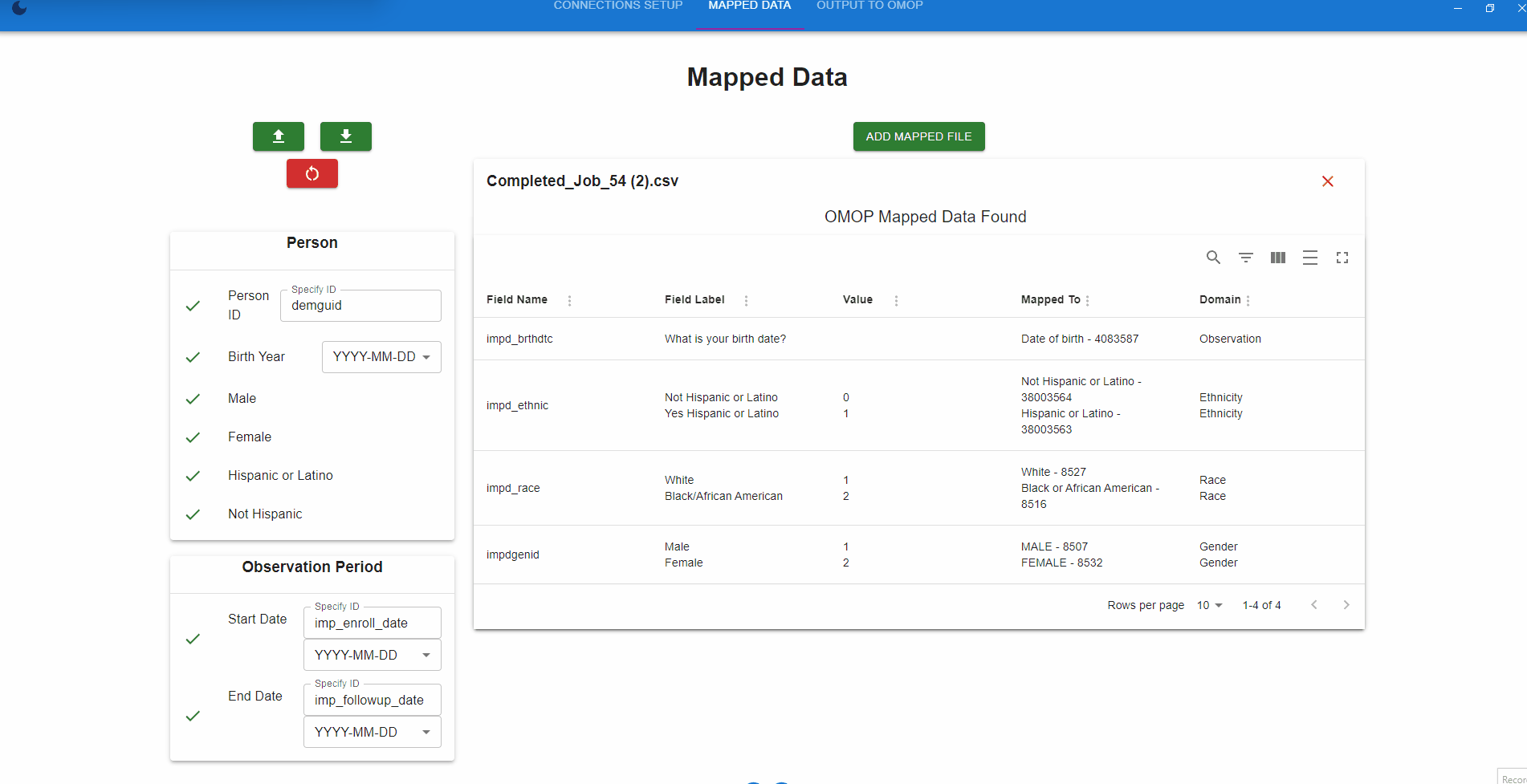

Supplement: Multimedia Appendix 2 [file jmir_v27i1e69004_app2.zip › IMPOWR-R24-REDCap-to-OMOP-main/frontend/src/assets/readme/desktopapp_output.gif]

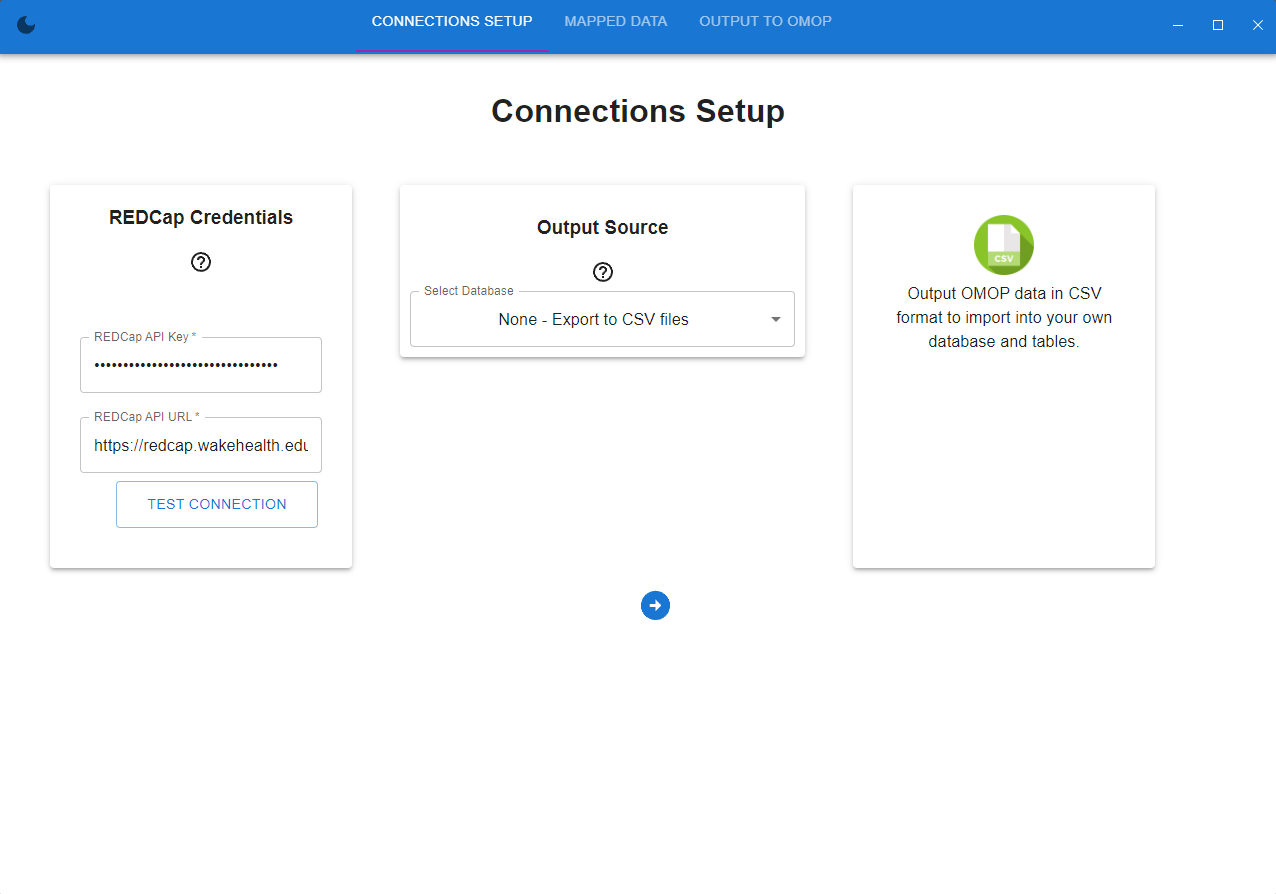

Supplement: Multimedia Appendix 2 [file jmir_v27i1e69004_app2.zip › IMPOWR-R24-REDCap-to-OMOP-main/frontend/src/assets/readme/desktopapp_setup_connection.png]

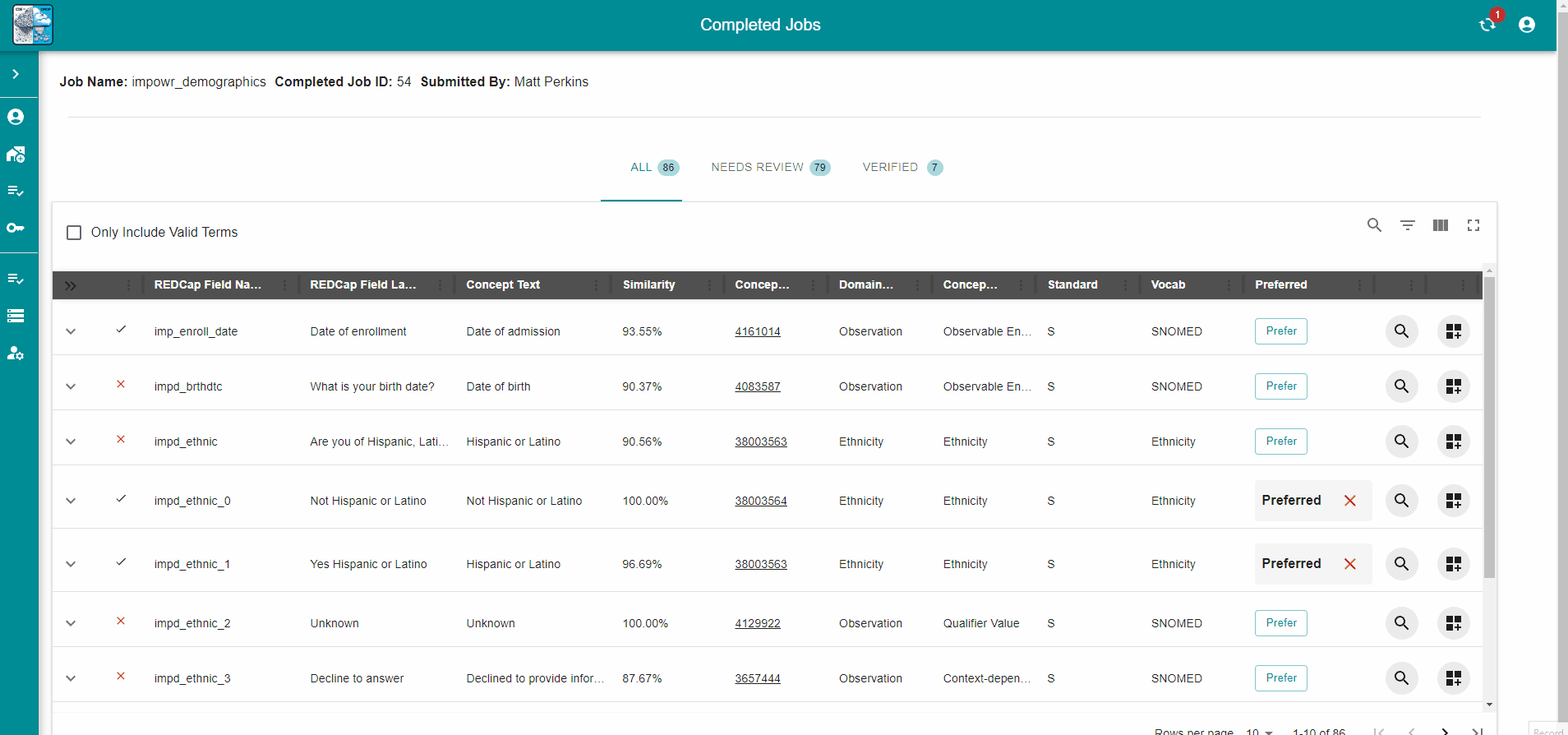

Supplement: Multimedia Appendix 2 [file jmir_v27i1e69004_app2.zip › IMPOWR-R24-REDCap-to-OMOP-main/frontend/src/assets/readme/webapp_download.gif]

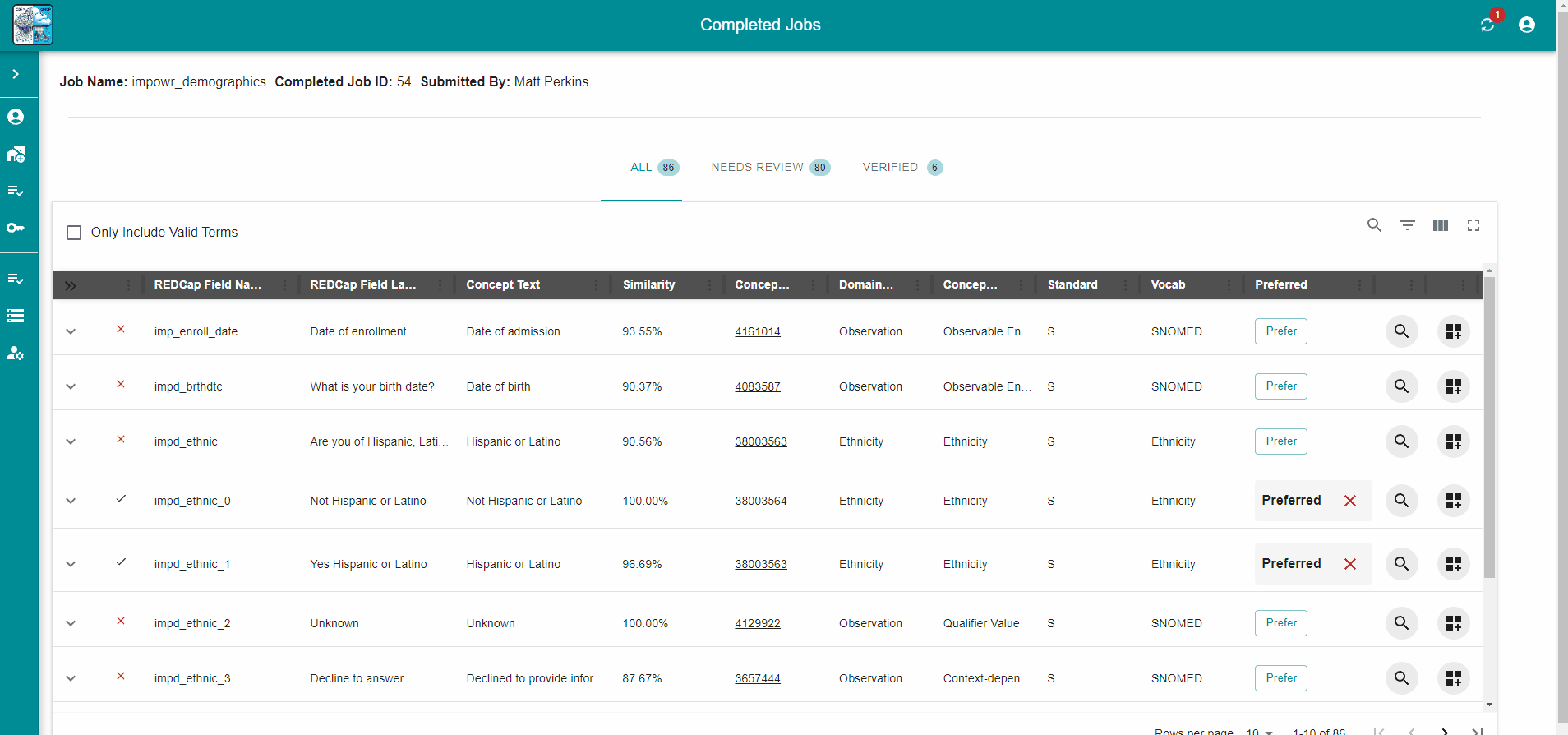

Supplement: Multimedia Appendix 2 [file jmir_v27i1e69004_app2.zip › IMPOWR-R24-REDCap-to-OMOP-main/frontend/src/assets/readme/webapp_prefer.gif]

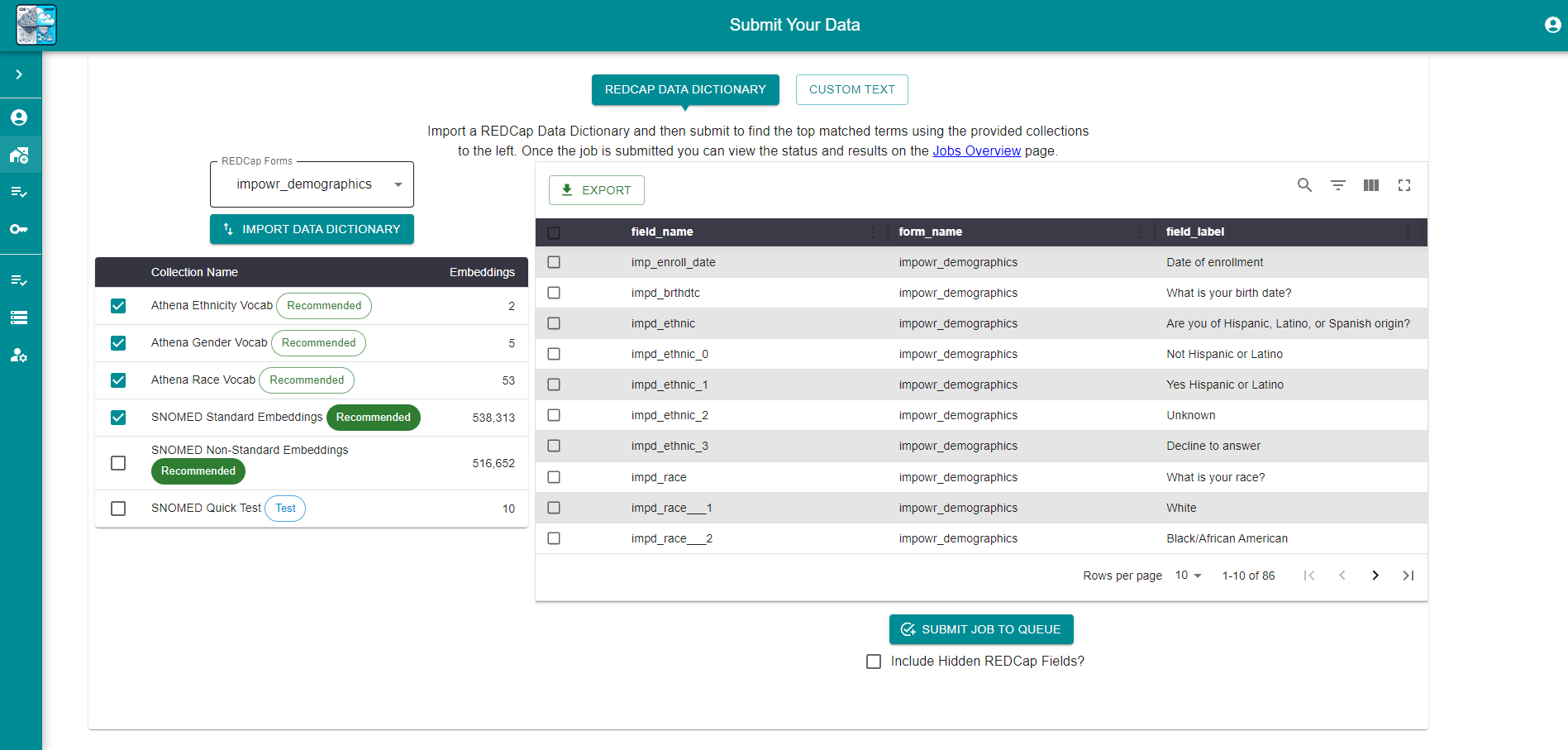

Supplement: Multimedia Appendix 2 [file jmir_v27i1e69004_app2.zip › IMPOWR-R24-REDCap-to-OMOP-main/frontend/src/assets/readme/webapp_submitData.png]

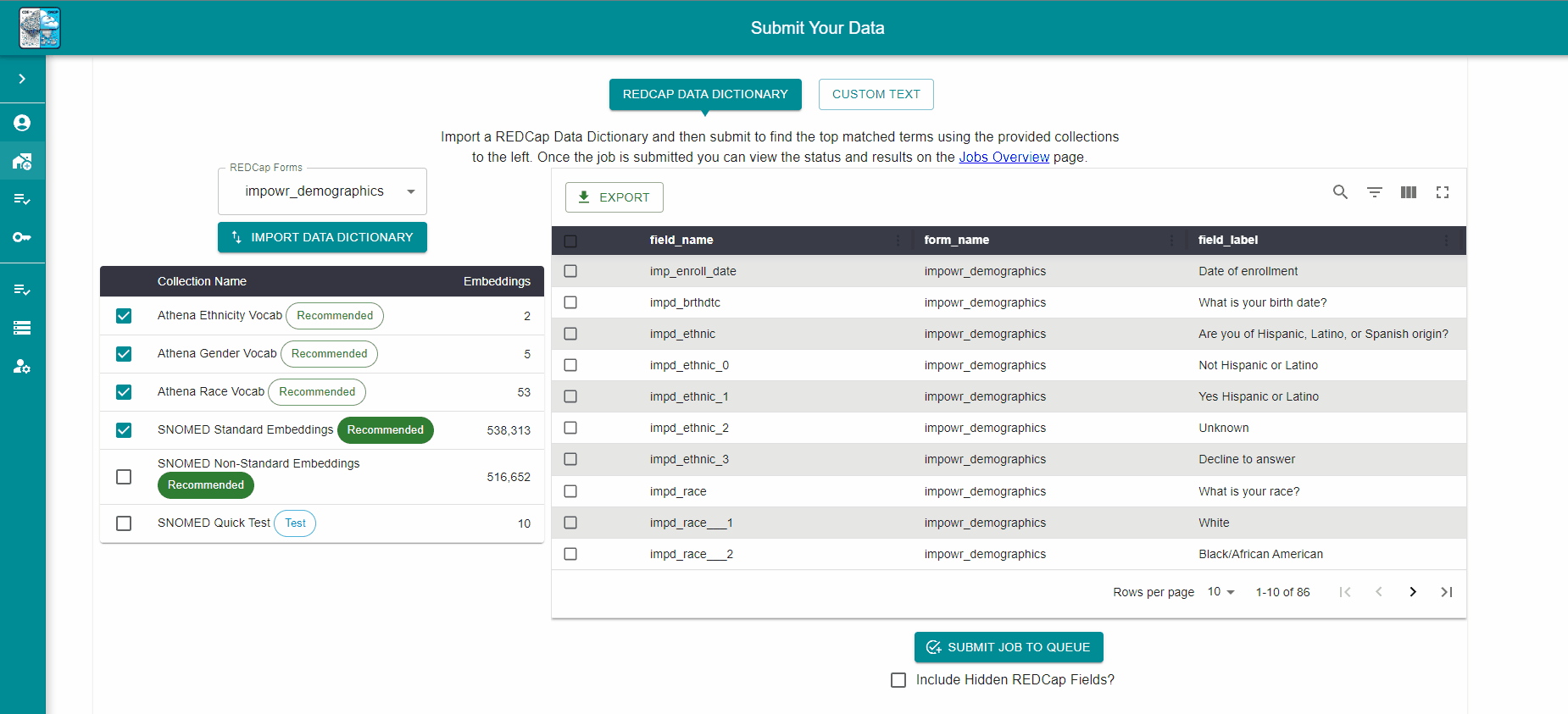

Supplement: Multimedia Appendix 2 [file jmir_v27i1e69004_app2.zip › IMPOWR-R24-REDCap-to-OMOP-main/frontend/src/assets/readme/webapp_submit_job.gif]

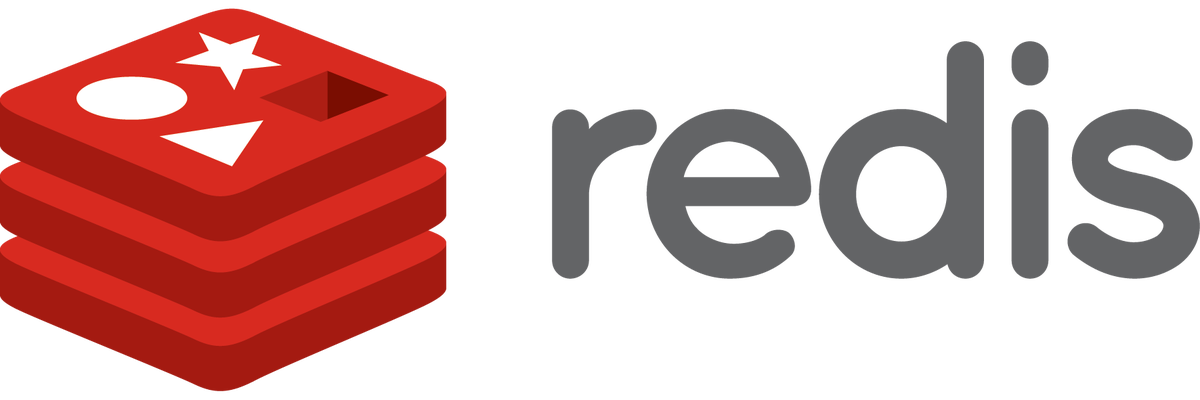

Supplement: Multimedia Appendix 2 [file jmir_v27i1e69004_app2.zip › IMPOWR-R24-REDCap-to-OMOP-main/frontend/src/assets/redis.png]

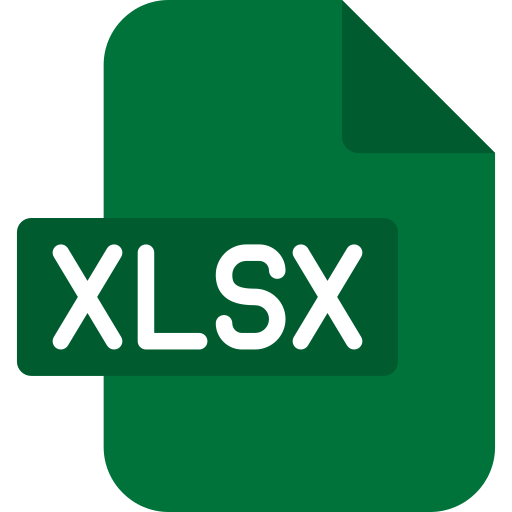

Supplement: Multimedia Appendix 2 [file jmir_v27i1e69004_app2.zip › IMPOWR-R24-REDCap-to-OMOP-main/frontend/src/assets/xlsx.png]
