# Supplementary material for: Breaking Digital Health Barriers Through a Large Language Model–Based Tool for Automated Observational Medical Outcomes Partnership Mapping: Development and Validation Study
Source: J Med Internet Res. 2025 May 15;27:e69004. doi: 10.2196/69004 (PMC12123247; doi:10.2196/69004)

REDCap to OMOP

Transforming healthcare data to fit the Observational Medical Outcomes Partnership (OMOP) Common Data Model (CDM) is essential for utilizing the Observational Health Data Sciences and Informatics (OHDSI) web tools, but it's a complex and daunting task. We begin with Athena, a resource aimed at mapping various terms to their corresponding OMOP tables. The challenge here is mapping your data, which could be derived from standard or non-standard terms from vocabularies like SNOMED or LOINC, to these Athena terms.

Traditionally, methods such as text comparison, fuzzy search, or tracking and weighting previously mapped values might be employed. However, we sought a more innovative solution by developing a single React and Node.js web app that would support multiple users and leverage the OpenAI GPT-3 word embeddings API. This approach involves storing terms from vocabularies like SNOMED in MongoDB collections. With this setup, users can retrieve their REDCap dictionary (or custom text) to be parsed and matched against selected word embedding collections.

The process involves sending text or survey data to the GPT-3 word embeddings API (unless already stored) and saving the resulting embeddings in a separate MongoDB collection. We then employ the cosine similarity function to ascertain the similarity between embeddings, generating a list of top matches. It's important to note here that this implementation is relatively slow and could be optimized with different techniques, particularly in the data storage and comparison techniques.

Once the matching process concludes, the web app presents an interface for users to review and select the most appropriate terms for their data. If a direct match isn't found, users can conduct a custom search in the Unified Medical Language System (UMLS). The final step involves exporting these mappings to a CSV companion file, which is then imported into a companion desktop app designed for building OMOP tables and performing the Extract, Transform, Load (ETL) process.

Choosing a desktop app for the ETL process was driven by the need to handle Protected Health Information (PHI) from REDCap securely. This setup facilitates managing security and user roles more effectively than a web app could, by limiting access to a single user per desktop app instance.

However, it's crucial to note that OMOP tables require a minimum amount of data to be mapped for successful ETL. At a minimum, the 'person' and 'observation_period' tables must be populated with the necessary fields. The desktop app must be informed about unique identifiers for individuals in the dataset and understand the date formats used. Once these prerequisites are met, users can proceed with the ETL, choosing between CSV or SQL files for their output and selecting the desired OMOP tables for their analysis.

Process Overview

1. Import REDCap Data Dictionary

The process begins with the importation of a REDCap data dictionary into the web application.

- **Step**: Import your data dictionary file.

- **Action**: Navigate to the import section and select your file.

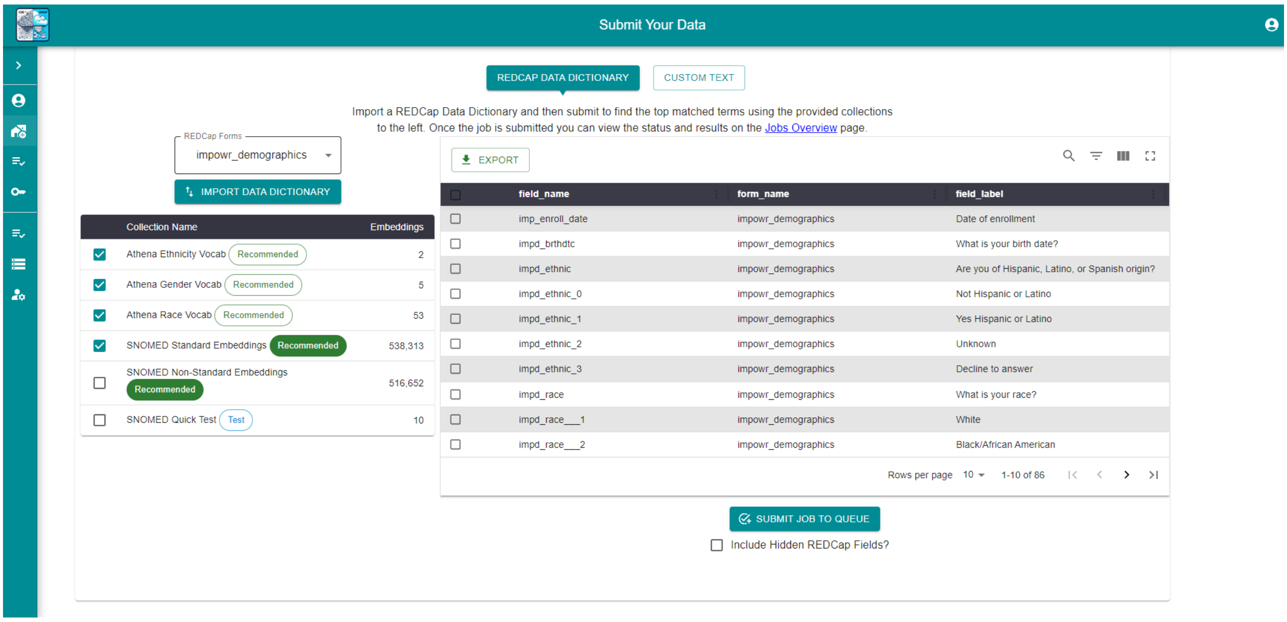

2. Data Mapping

Submitted data dictionaries undergo a mapping process against a MongoDB collection of SNOMED terms converted to word embeddings.

- **Step**: Submit the data dictionary for mapping.
- **Action**: Click the "Submit" button.

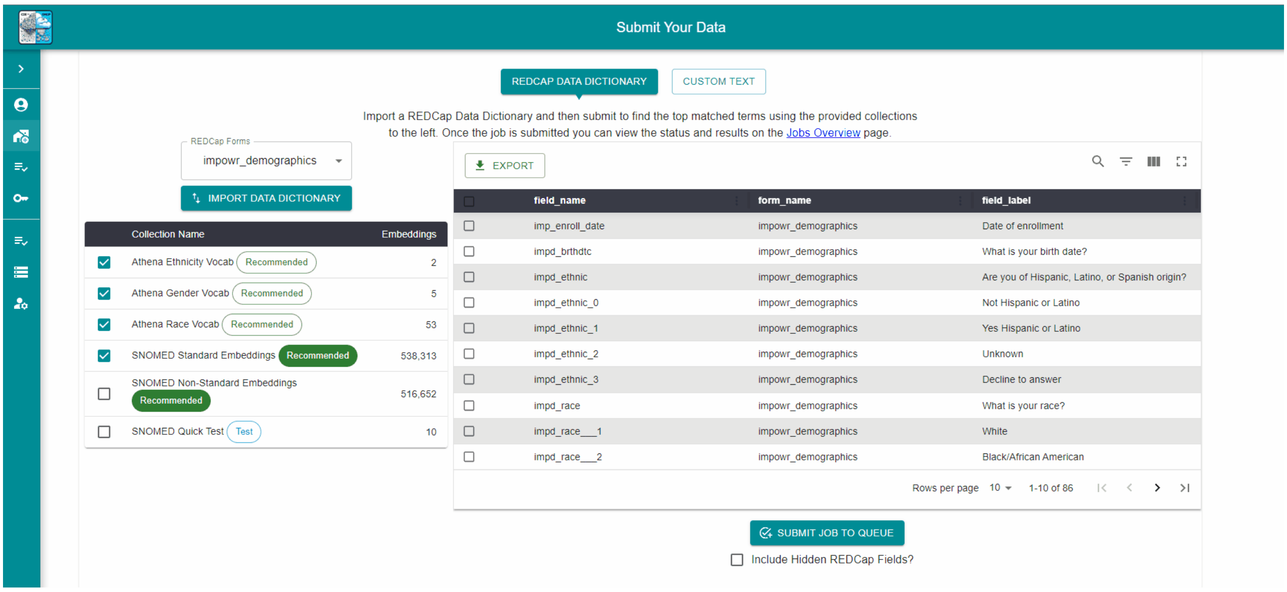

3. Mapping Review and Selection

Users review the suggested mappings in a table format and select the most accurate matches.

- **Step**: Review and select correct mappings.

- **Action**: Select the correct mapping for each field.

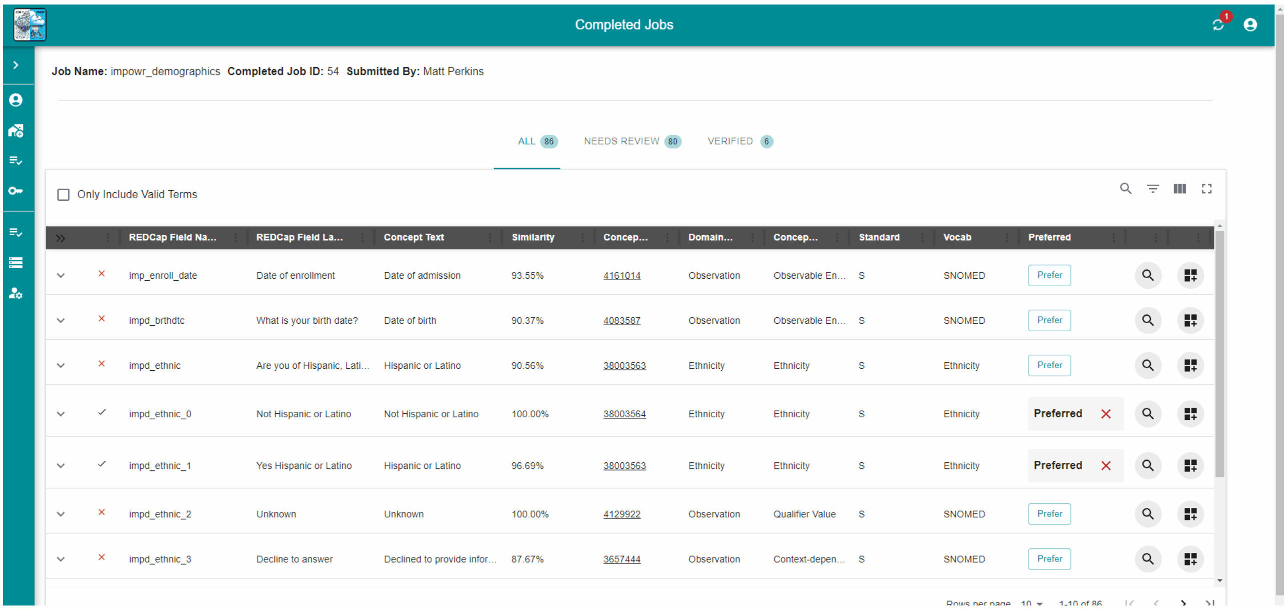

4. Download REDCap Companion File

Finally, users can download a companion file with their selected mappings added to the field_annotation column.

- **Step**: Download the companion file.
- **Action**: Click on the "OMOP Companion File" icon/button.

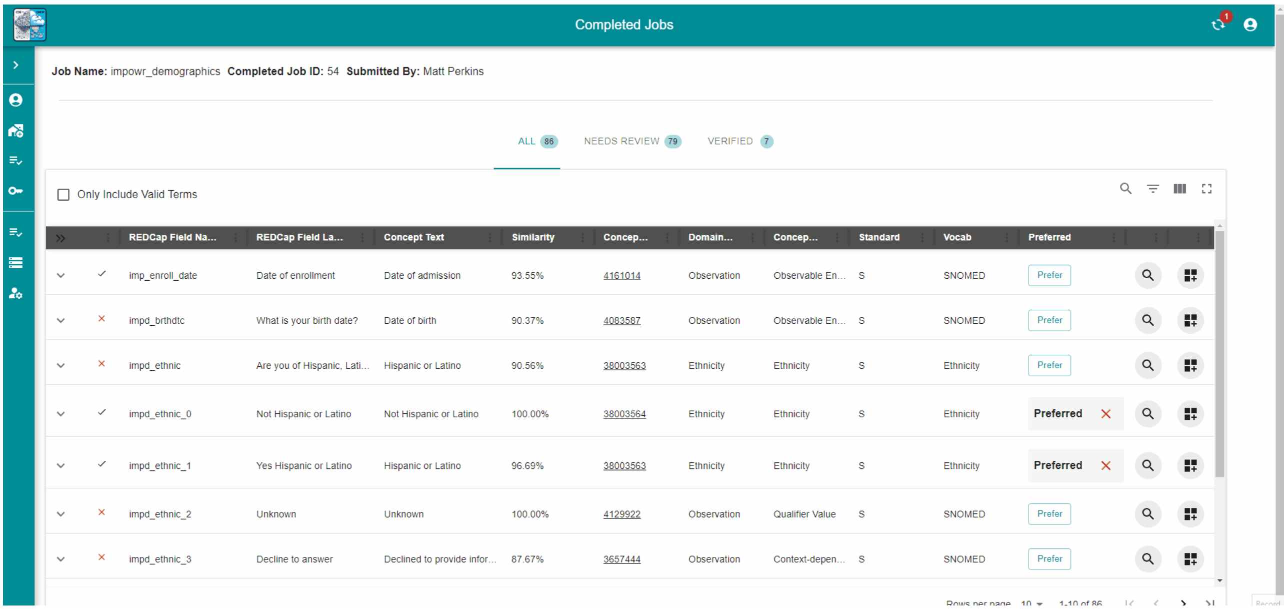

Desktop Electron.js App Workflow

1. Setup and Configuration

Users set up their REDCap connection and add the companion file in the desktop app.

- **Step**: Configure REDCap connection

- **Action**: Through the app's settings, configure the connections

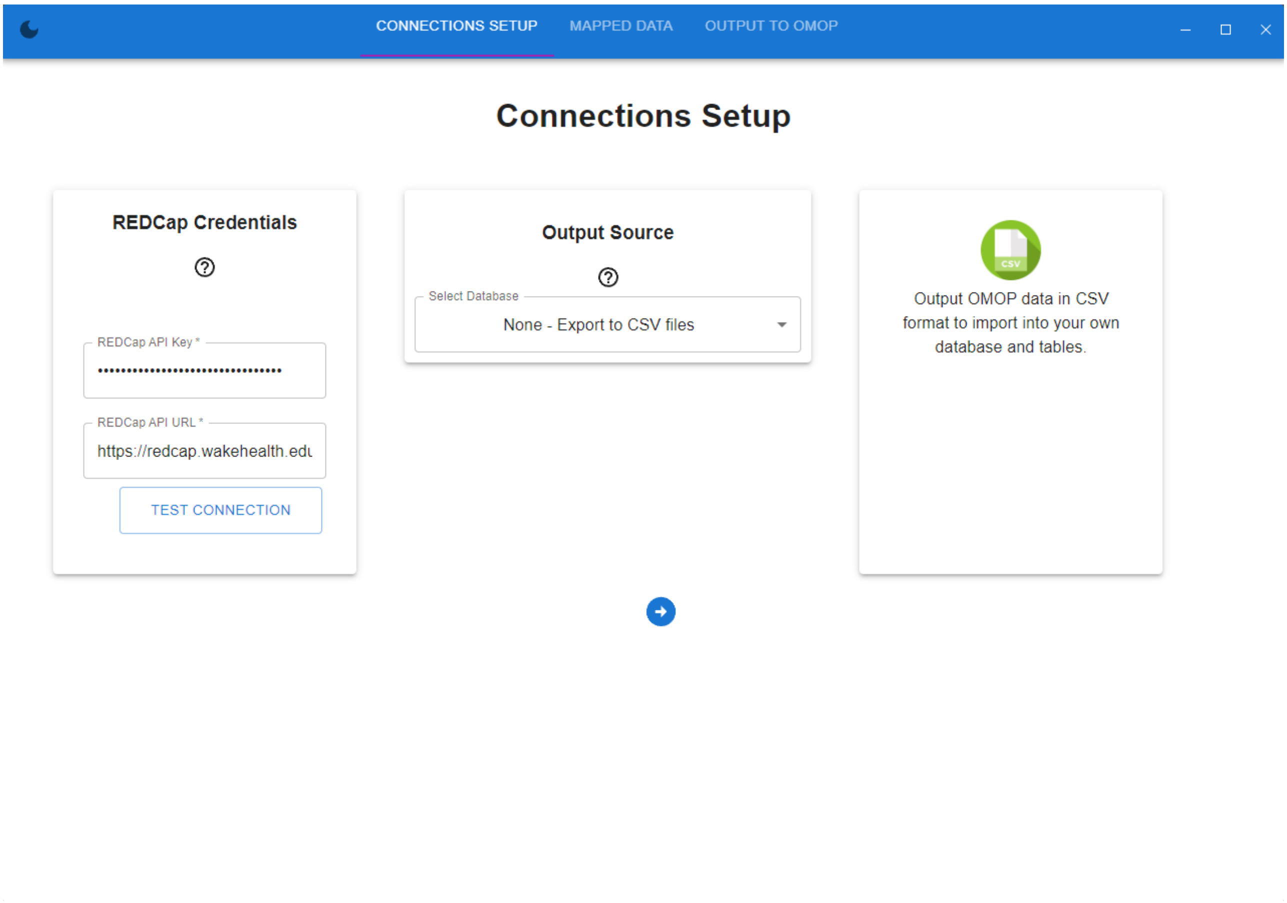

2. Add the companion file

The desktop app uses the mapped data that is linked to the REDCap data dictionary. Additional info is required such as Person ID, date formats, and the field names for start and end dates.

- **Step**: Add companion file.
- **Action**: Click "Add Mapped File" and select the downloaded companion file from the web app.

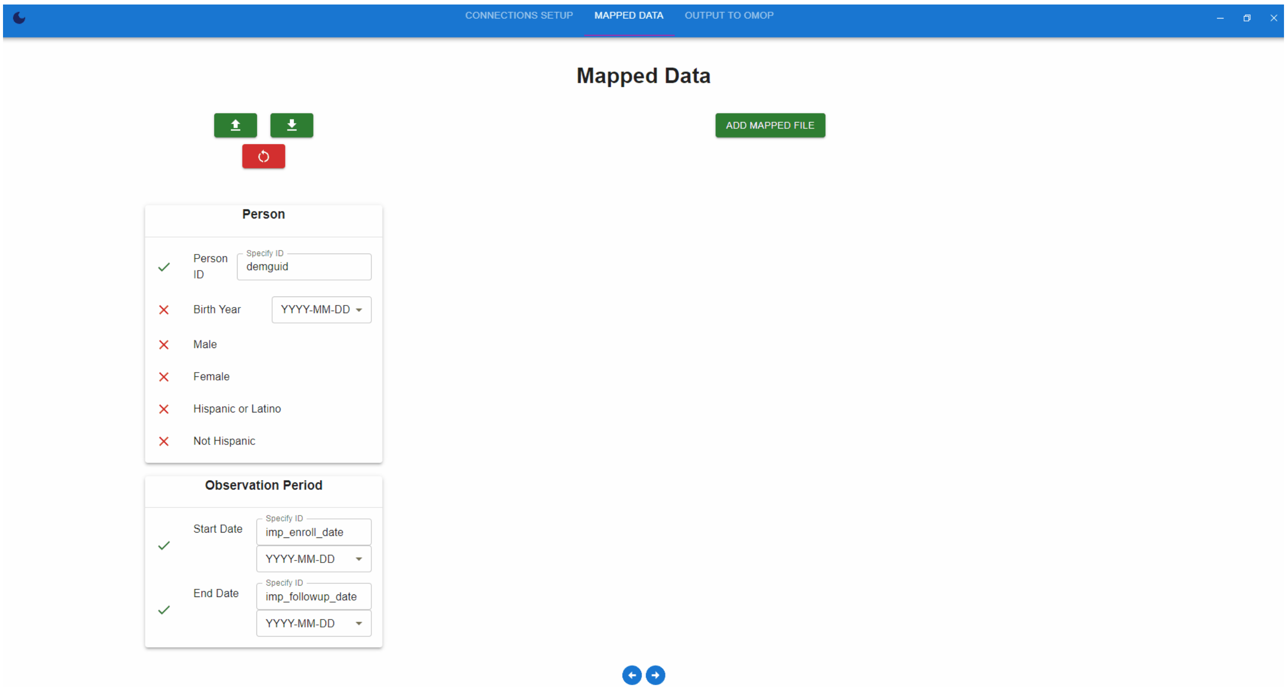

3. Data Extraction, Transformation, and Loading (ETL)

The desktop app uses the mapped data to extract records from REDCap, transform them according to OMOP standards, and load them into the chosen database format.

- **Step**: Perform ETL on mapped data.
- **Action**: Choose the output format (SQL or CSV) and execute the ETL process.

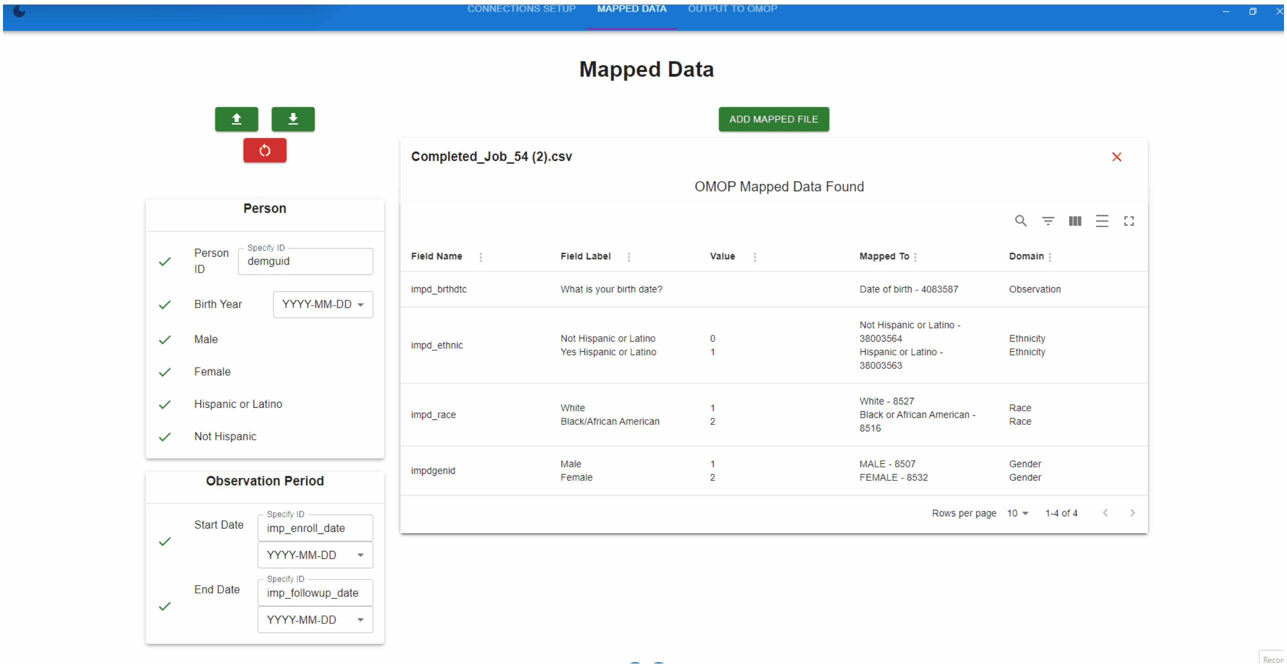

Supplement: Multimedia Appendix 3 [file jmir_v27i1e69004_app3.pdf]
